# Single-cell transcriptomic profiling reveals diversity in human iNKT cells across hematologic tissues

**DOI:** 10.1016/j.celrep.2025.115587

**Published:** 2025-04-28

**Authors:** Reyka G. Jayasinghe, Derek Hollingsworth, Nathan C. Schedler, Emily Landy, Chaiyaporn Boonchalermvichian, Biki Gupta, Hao Yan, Jeanette Baker, Beruh Dejene, Kenneth I. Weinberg, Robert S. Negrin, Melissa Mavers

**Affiliations:** 1 Department of Medicine, Division of Oncology, Washington University School of Medicine, Saint Louis, MO, USA; 2 Department of Pediatrics, Division of Hematology, Oncology, Stem Cell Transplantation and Regenerative Medicine, Stanford University School of Medicine, Stanford, CA, USA; 3 Department of Pediatrics, Division of Hematology and Oncology, Washington University School of Medicine, St. Louis, MO, USA; 4 Department of Medicine, Division of Blood and Marrow Transplantation and Cellular Therapy, Stanford University School of Medicine, Stanford, CA, USA; 5 These authors contributed equally; 6 Present address: Insitro, South San Francisco, CA, USA; 7 Present address: Wake Forest University School of Medicine, Winston-Salem, NC, USA; 8 Present address: Ohio University, Heritage College of Osteopathic Medicine, Beachwood, OH, USA; 9 Lead contact

## Abstract

Jayasinghe et al. provide a comprehensive transcriptional analysis of human invariant natural killer T cells from multiple immunologically relevant hematologic tissues, revealing naive/precursor, transitional, and Th1/17/NK-like cells. Through single-cell RNA sequencing and flow cytometry, they highlight both unique aspects of human iNKT cell biology distinct from mouse cells and similarities.

## SUMMARY

Invariant natural killer T (iNKT) cells are evolutionarily conserved innate lymphocytes important for protection against pathogens, malignancies, and graft-versus-host disease, with potential for universal donor cellular therapies. While mouse studies reveal transcriptionally and functionally distinct subsets, a comprehensive understanding of human iNKT cell heterogeneity is limited. Herein, we delineate the transcriptomic diversity of human iNKT cells from multiple immunologically relevant hematologic tissues. Human iNKT cells express naive/precursor, transitional, and T helper (Th)1/17/NK-like transcriptional profiles, partially contrasting with findings in mice. Additionally, these data uncover transcription factor dynamics not previously described in mice and reveal a T effector memory RA^+^-like population. Further, two distinct expression patterns of human CD8^+^ iNKT cells are described—one resembling naive/precursor cells and another resembling Th1/17/NK-like cells, with predominant expression of CD8*αα* protein. These critical insights into the transcriptional heterogeneity of human iNKT cells will facilitate future functional studies and inform iNKT-based cellular therapy development.

## Graphical abstract

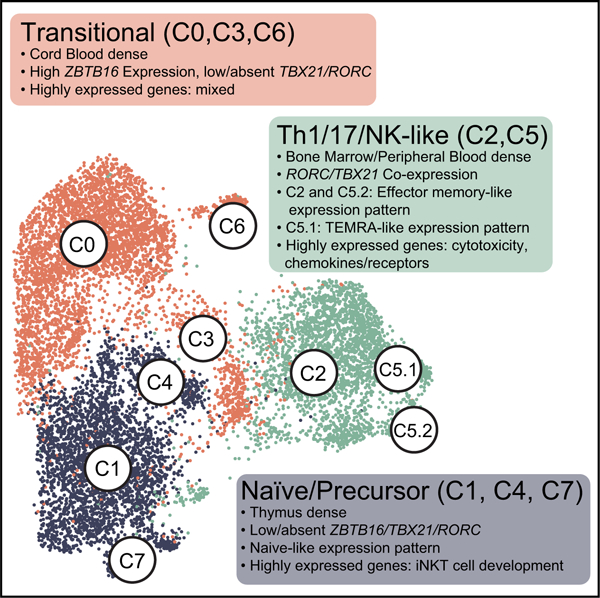

## INTRODUCTION

Invariant natural killer T (iNKT) cells lie at the interface of innate and adaptive immunity, playing important protective roles in responses to pathogens and surveillance for malignant cells. iNKT cells recognize glycolipid antigen via their invariant T cell receptor (TCR; Vα24Jα18/Vβ11 in humans and Vα14Jα18 with Vbβ, −7, or −8 in mice^1,2^) when presented by the major histocompatibility complex (MHC)-like protein CD1d.^3^ As CD1d is monomorphic, iNKT cells are incapable of allorecognition and unable to initiate graft-versus-host disease (GVHD), making them a powerful platform for universal donor cellular therapies. Clinical trials of allogeneic chimeric antigen receptor (CAR)-engineered iNKT cells for cancer therapy are underway (ClinicalTrials.gov: NCT03774654, NCT05487651, and NCT06182735).

While iNKT cells are not capable of causing GVHD, substantial evidence suggests that they can suppress it.^4^ The adoptive transfer of donor or third-party iNKT cells into mouse models of acute and chronic GVHD provides protection from disease.^5–8^ A conditioning regimen that facilitates relative retention of host iNKT cells is associated with GVHD protection in both mice and humans.^9–12^ Higher iNKT cell numbers in hematopoietic stem cell transplantation (HSCT) grafts,^13^ or more rapid iNKT cell reconstitution post-transplant,^14,15^ are associated with reduced GVHD as well.

Mouse iNKT cell development has been described to progress linearly along stages 0–3. Stage (ST) 0 cells (CD24^+^CD44^−^ NK1.1^−^) represent the earliest detectable iNKT cells in the thymus and undergo progressive loss of CD24 (ST1), expression of CD44 (ST2), and expression of NK1.1 in the most mature ST3 cells, with thymic egress occurring primarily in ST2.^2,16,17^ Functionally distinct subsets of mouse iNKT cells similar to T helper (Th) cell subsets have been reported, with Th1-like iNKT1 cells exclusively expressing T-bet and producing primarily interferon (IFN)γ, Th2-like iNKT2 cells expressing the highest levels of promyelocytic leukemia zinc finger (PLZF) and interleukin (IL)-4, and Th17-like iNKT17 cells exclusively expressing RAR-related orphan receptor-gamma t isoform (RORγt) and IL-17.^2,18–20^ Attempts to integrate these paradigms indicate that iNKT2 cells overlap with ST1–2 cells, iNKT1 and iNKT17 cells separately differentiate from iNKT2 cells, and iNKT1 cells overlap with ST3 cells, suggesting that some ST1–2/iNKT2 cells are a transitional population between precursors and fully differentiated iNKT cells.^21,22^ However, linear development through these stages has been questioned, with one study showing that Th2-like iNKT cells cannot give rise to iNKT1 cells.^23^ Functionally, iNKT2 and iNKT17 cells suppress acute GVHD in mouse models, while iNKT1 cells exhibit strong cytotoxicity toward malignant cells.^18^ These results suggest that the alignment of particular subsets with the clinical goal of iNKT-based cellular therapies may enhance safety and efficacy. However, a better understanding of human iNKT cell subsets is required to realize their full clinical potential, and a comprehensive study of human iNKT cell heterogeneity with robust cell numbers from immunologically relevant hematologic tissues is lacking.

Herein, we define the transcriptomic heterogeneity of human iNKT cells from peripheral blood (PB), cord blood (CB), bone marrow (BM), and thymus. Because future therapeutic use of distinct subsets would require distinguishing these subsets in an unstimulated state, we studied the heterogeneity of unstimulated human iNKT cells. While identifying many of the same populations described previously in mice, we report several differences, including a mixed Th1/17 signature, two transcriptionally distinct CD8^+^ iNKT cell clusters, and a CD4^−^CD8^−^ T effector memory (T_EM_) RA^+^ (TEMRA)-like population. These data define the transcriptional heterogeneity of human iNKT cells and provide a critical resource to build our understanding of human iNKT cell subsets.

## RESULTS

### Survey of the human iNKT cell landscape at single-cell resolution

**Figure 1.**
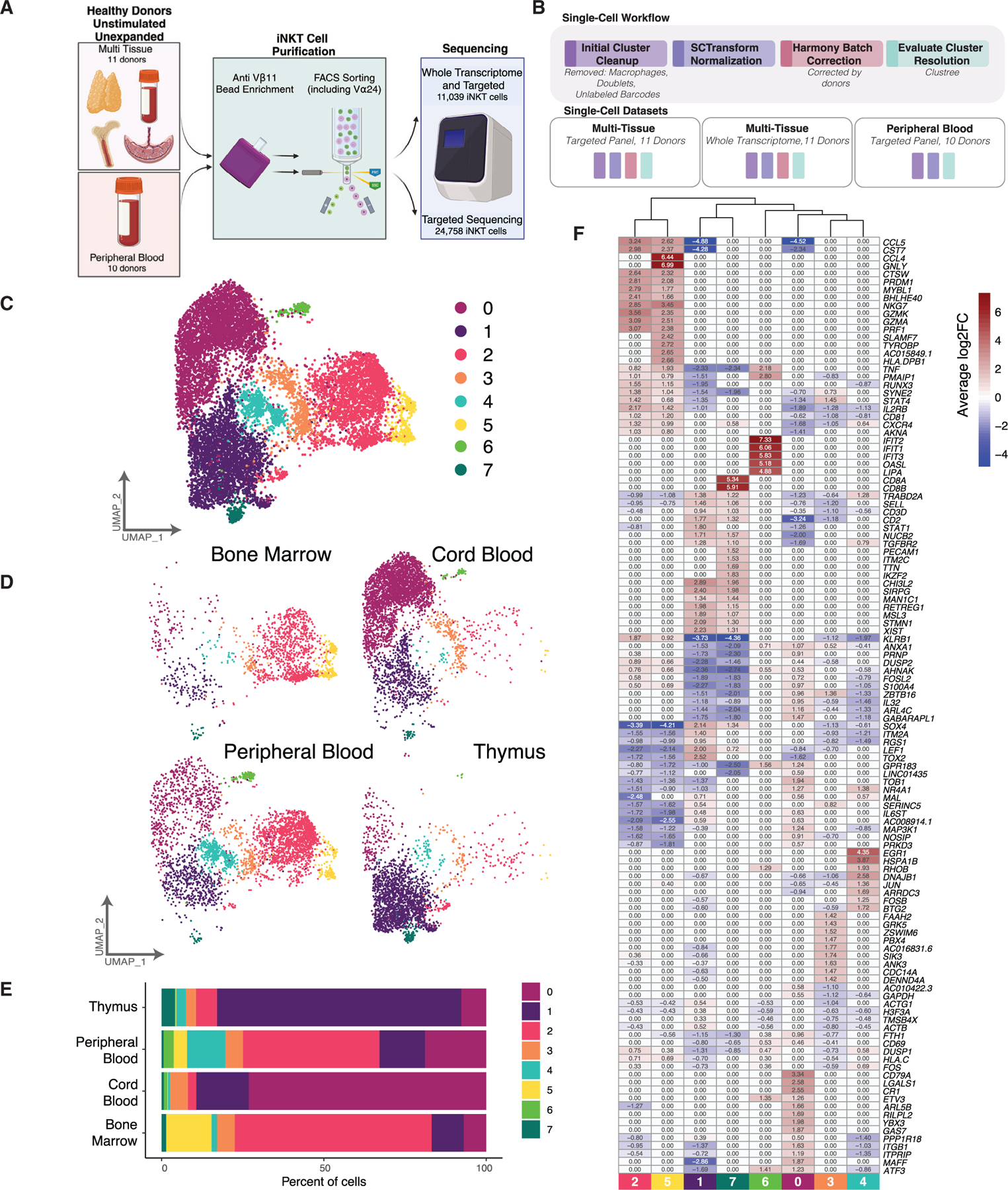
Overview of scRNA-seq datasets generated from human iNKT cells (A) Schematic of iNKT cell purification and sequencing. (B) Overview of single-cell workflow for each dataset. (C) UMAP of all iNKT cells in the multi-hematologic tissue cohort. Cells are colored by cluster assignment. (D) UMAP representation of all iNKT cells separated by tissue source. Cells are colored by cluster assignment. (E) Bar plot indicating proportions of iNKT cells restricted to each cluster separated by tissue source. (F) Heatmap of top 10 differentially expressed genes identified for each cluster. Each cell is colored by the average log2 fold change (log2FC) of each gene identified in each cluster (0 indicates the gene is not differentially expressed). See also Figure S1 and Tables S1, S2, and S3.

The transcriptional heterogeneity of human iNKT cells was investigated in two experiments, one with 11 unpaired donors of PB (*n* = 3), CB (*n* = 3), thymus (*n* = 3), and BM (*n* = 2) and another with PB only (*n* = 10) (Figures 1A and 1B). iNKT cells were enriched by magnetic beads and sorted by fluorescence-activated cell sorting (FACS) using antibodies to the invariant TCR, including the removal of CD24^+^ ST0 iNKT cells to ensure adequate numbers of differentiating and mature thymic iNKT cells to better facilitate comparison across tissues. Following library generation and sequencing, 11,039 cells underwent whole transcriptome analysis (WTA) in the multi-hematologic tissue experiment after passing quality control and the removal of a small number of contaminating cells, including macrophages (highly expressing *FCER1G*, *FCGR3A*, *LYN*, and *IL1B*) and conventional T cells (expressing an α or β chain different than the invariant TCR, using VDJ sequencing) (Table S1). This experiment also utilized targeted single-cell RNA sequencing (scRNA-seq) with a 435 gene panel custom designed to include genes important for NK cell and T cell function, including both proinflammatory and immune regulatory subsets (Table S2). The PB-only experiment underwent targeted sequencing only, with 24,758 cells passing quality control. Oligomer-conjugated antibodies were used to detect CD45RA, CD45RO, CD4, and CD8.

Transcriptional heterogeneity of human iNKT cells from PB, CB, thymus, and BM revealed eight iNKT cell clusters (Figure 1C). While iNKT cells from some tissue sources dominated certain clusters, substantial numbers of PB-derived iNKT cells were detected in all clusters (Figures 1D and 1E). These findings persisted across individual donors (Figures S1A and S1B). The top 10 differentially expressed genes between clusters revealed distinguishing features (Figure 1F; Table S3).

### Patterns of *TBX21* and *RORC* expression in human iNKT cells reveal Th1/17-like cells not described in mice

**Figure 2.**
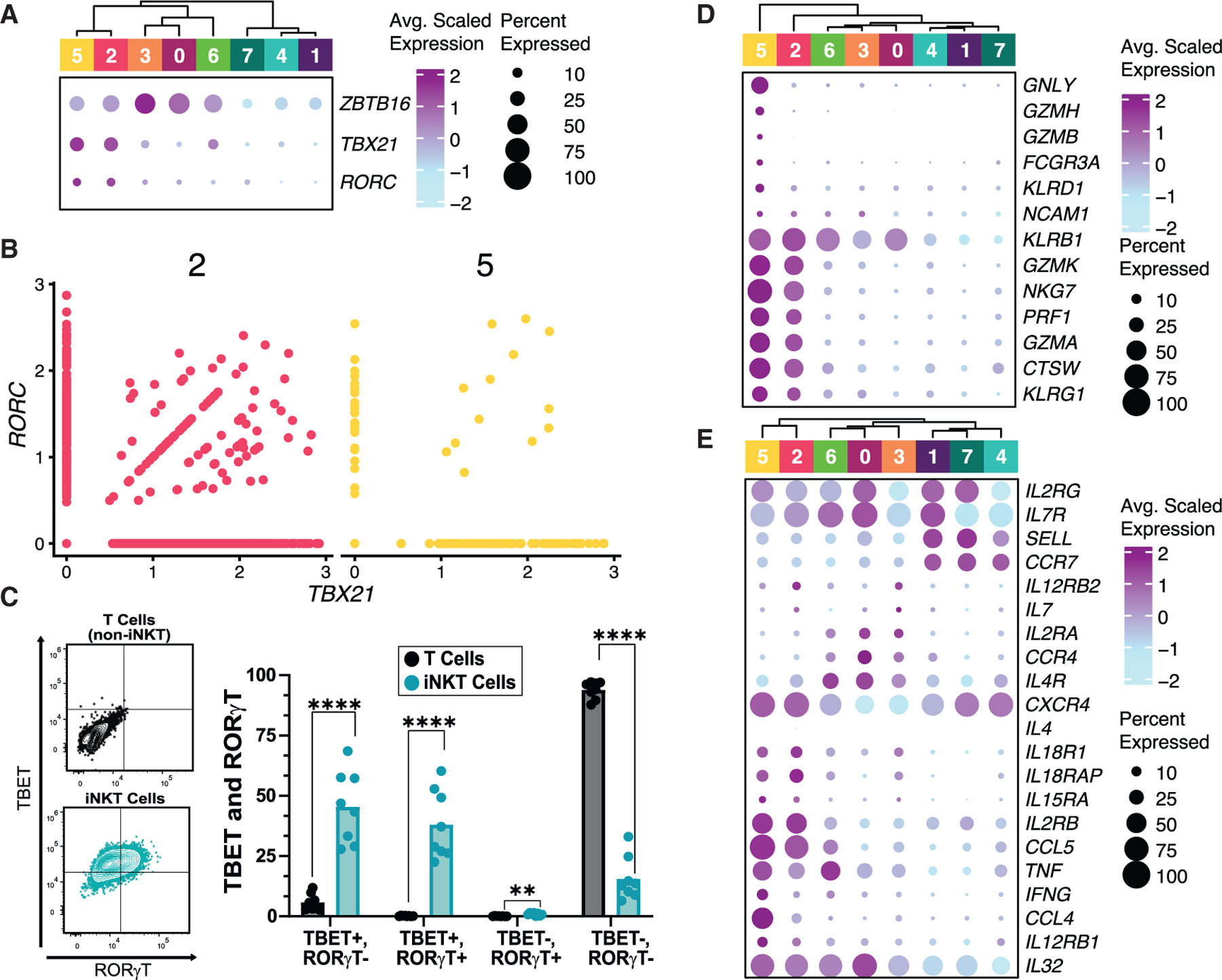
Human iNKT cell transcriptional heterogeneity reveals Th1/17/NK-like cells, transitional cells, and early precursor cells (A) Expression of key transcription factors. Size and color indicate the percentage of cells expressing a gene and the average scaled expression, respectively. (B) Scatterplot of cells exhibiting co-expression of *RORC* and *TBX21*. Each plot is separated (and colored) by cells derived from clusters 2 and 5. (C) Representative flow cytometry dot plot (left) and summary flow data (right) of Tbet and RORγt protein expression in PB iNKT cells after 72 h stimulation. Bar graphs represent the mean, with individual donor data points plotted. **p < 0.001 and ****p < 0.0001 by paired t tests followed by Holm-Šídák multiple comparisons test. Data were pooled from at least three independent experiments. (D and E) Expression of a subset of genes. Size and color indicate the percentage of cells expressing a gene and the average scaled expression, respectively. See also Figures S1 and S3.

We sought to evaluate the expression patterns of well-described transcription factors that define mouse iNKT cell functional subsets.^2,18–20^ In contrast to mouse cells, clusters of human iNKT cells that express *RORC*, including cluster 2 (C2) and C5 (both predominantly BM derived), display co-expression of *TBX21* (Figures 2A, 2B, and S1C). Tbet^+^RORγt^+^ iNKT cells were also detected by flow cytometry in human PB, though Tbet^+^RORγt^−^ iNKT cells were detected as well (Figures 2C, S1D, and S1E). This combined Th1/17 signature has been previously described for human mucosal-associated invariant T (MAIT) cells.^24^ Three thymic-dominant clusters, C1, C4, and C7, had minimal expression of *TBX21* and *RORC* and retained expression of *ZBTB16*, albeit with somewhat lower expression than in other clusters (Figure 2A), similar to findings in post-ST0 early iNKT precursor cells in mice.^22^ C0 and C3 (CB dominant) both had high expression of *ZBTB16* and low/absent expression of *TBX21* and *RORC* relative to other clusters, while C6 co-expressed *ZBTB16* and low levels of *TBX21*, suggesting these clusters represent a progressive transition between precursor iNKT cells and Th1/17-like iNKT cells (Figure 2A). This correlates with mouse data demonstrating transitional populations between early precursor iNKT cells and iNKT1 cells, which have some overlap with iNKT2 cells.^21,22^ Collectively, these results suggest that human iNKT cells can be described as early precursor, transitional, or Th1/17-like cells based on their expression patterns of transcription factors canonically defining mouse iNKT cell subsets.

To contextualize our findings within previous research, we integrated our human iNKT cell data with a published mouse iNKT cell transcriptional dataset (Figures S2A–S2C).^25^ Despite excluding CD24^+^ cells, human thymic-derived iNKT cells primarily aligned with mouse ST0 thymic iNKT cells, further supporting an early precursor signature. Human CB-derived cells (C0 and C3 transitional clusters) align with mouse thymic ST1–3. Human BM-derived cells (and most PB-derived cells) align primarily with mouse thymic ST3 and peripheral tissue-derived iNKT cells. These data support a human iNKT cell developmental trajectory similar to that described for mice.^21,22^ We also integrated our data with a recently published transcriptome dataset that included human thymic-derived MAIT cells, conventional T cells, and NKT cells (Figure S2D).^24^ Thymic-derived C1 largely overlaps with the immature and interim MAIT, NKT, and naive conventional T cells from the prior analysis. C2 and C5 overlap with their MAIT 1/17 and iNKT 1/17 cells, suggesting that a combined Th1/17 signature is conserved across various human innate lymphocytes. Altogether, this alignment of our data allows for comparing mouse and human iNKT cells, using reference populations to better understand the scale of differences between human iNKT cell clusters, and identifying similar transcriptional signatures among human innate lymphocytes.

### Molecular expression signatures suggest distinct effector functions between iNKT cell clusters

Mouse iNKT1 cells possess cytotoxic capabilities that are absent in other iNKT subsets.^18^ To investigate this in human iNKT cells, we interrogated the expression pattern of genes important for cytotoxicity and other NK cell functions, finding them significantly increased in the Th1/17-like C2 and C5 relative to other clusters (Figure 2D). This included high expression of *GZMK* in most C2 and C5 cells, with a very small proportion of cells expressing *GZMB* in C5 only (*GZMK*, C2: 3.56 and C5: 2.35 average log2 fold change [avg log2FC]), similar to many cyto-toxic innate lymphocytes.^26^ We also observed increased expression of *KLRG1* and *GZMA* in these two clusters, similar to a KLRG1^+^GZMA^+^ population of long-lived iNKT cells deriving from iNKT1 cells in mice (*KLRG1*, C5: 1.88 avg log2FC; *GZMA*, C2: 3.09 and C5: 2.51 avg log2FC).^27^ Expression of *GNLY* (gene encoding granulysin, a component of cytotoxic T and NK cell granules and a chemoattractant with the ability to induce inflammatory cytokines^28^) was strongly increased in C5 relative to other clusters (6.99 avg log2FC). *KLRB1* (gene encoding CD161, the human homolog of NK1.1 in C57BL/6 mice) is a marker of fully differentiated iNKT cells corresponding to iNKT1 cells.^29,30^ Accordingly, *KLRB1* expression arises in C0 and C6, with the highest expression in C2 and C5 (C2: 1.87 and C5: 0.92 avg log2FC). Overall, these results suggest that Th1/17-like clusters (C2 and C5) can be best described as Th1/17/NK-like. Despite this, less than 5% of cells express *NCAM1* (gene encoding CD56) in any cluster, though the expression is the highest in C2, C3, and C5 (Figure 2D). A previous report similarly found that very few human iNKT cells express CD56 protein.^31^

Detection of cytokines is notoriously low in scRNA-seq datasets, particularly in unstimulated cells, and *IL4* and *IFNG* were sparsely detected, with *IFNG* most highly expressed in C5 (Figures 2E and S3A). *TNF* expression was highest in C2, C5, and C6 (C2: 0.82, C5: 1.93, and C6: 2.18 avg log2FC). Cytokine receptor expression suggested functionally distinct profiles of cytokine responsiveness between clusters, including relatively increased expression of *IL18R1*, *IL18RAP*, *IL15RA*, and *IL12RB1* in C2 and C5 (Figure 2E). Expression of *IL2RB* (gene encoding IL-2Rβ, part of the intermediate and high-affinity IL-2 receptors (IL-2Rs) and the IL-15R) was also highly expressed in C2 and C5 (*IL2RB*, C2: 2.17 and C5: 1.42 avg log2FC). *IL4R* expression was highest in C0 (0.89 avg log2FC) and slightly decreased in C6 and C3 relative to C0, suggesting that these transitional cells may have some Th2-like features as described in mice^18,21,22^ and correlating with previous studies demonstrating that CB- and neonatal-derived iNKT cells have a Th2-skewed cytokine pattern.^32,33^ However, *IL17RB* expression was not detected (Figure S3A), as has been described in mouse Th2-like cells.^20,23,34^ Interestingly, C6 was characterized by high expression of *IFIT1*, *IFIT2*, and *IFIT3* (Figure 1F), indicative of a type I IFN response, with a similar cluster previously detected in several mouse studies.^21,25,35^ *CCL5* and *CXCR4* were the most highly expressed chemokine and chemokine receptor, respectively, and were strongly expressed in C2 and C5 relative to other clusters (*CCL5*, C2: 3.24 and C5: 2.62 avg log2FC; *CXCR4*, C2:1.32 and C5: 0.99 avg log2FC) (Figures 2E and S3A). CXCR4 promotes lymphocyte chemotaxis as the CXCL12/stromal-derived factor 1 receptor and is highly expressed in PB iNKT cells.^36^ CCL5 recruits immune cells to sites of inflammation and is upregulated in a subset of iNKT1 cells in mice.^21^ Collectively, these results support the Th1/17/NK-like signature of C2 and C5 and suggest that the transitional C0, C3, and C6 clusters may have some Th2-like features.

### Regulon analysis uncovers transcription factors active in distinct human iNKT cell subsets

**Figure 3.**
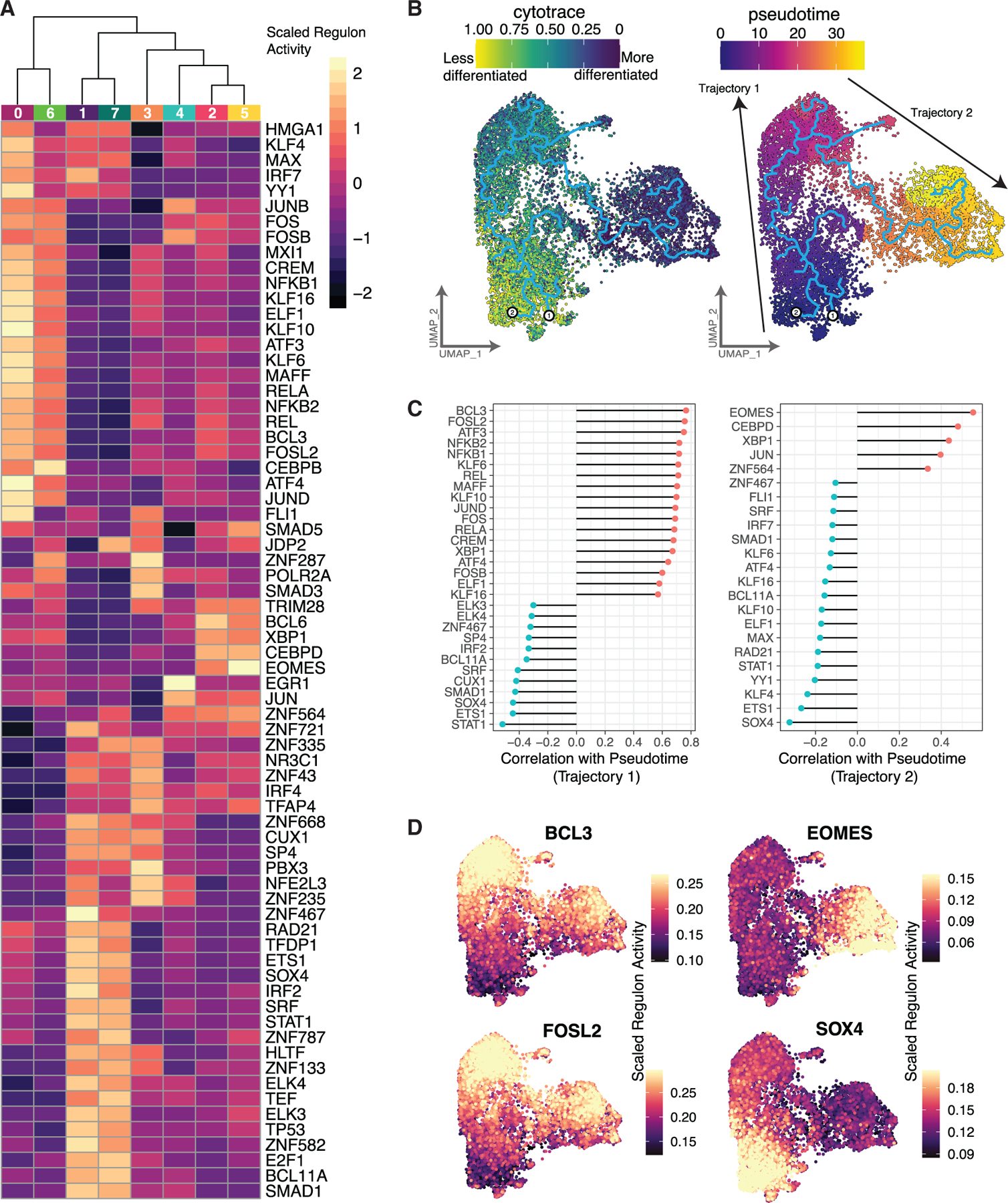
Transcription factor regulon activity between clusters and across pseudotime differentiation trajectories (A) Heatmap of all regulons identified across iNKT cell clusters. Each region is colored by the scaled regulon activity of all cells belonging to the annotated cluster. (B) UMAP of iNKT cells colored by CytoTRACE score (left) and pseudotime (right). Overlaying trace indicates predicted trajectories using Monocle3. Two arrows indicate annotated trajectories for the following panels. (C) Subset of regulons correlating with pseudotime across the annotated trajectory. For each gene, the resulting correlation value (after false discovery rate [FDR] correction) of the regulon with pseudotime is indicated. (D) UMAP representation of annotated regulon with each cell colored by SCENIC activity score of selected regulons from trajectories 1 and 2. See also Figure S4.

We next performed a regulon analysis, using SCENIC, to unveil additional transcription factors regulating distinct clusters of human iNKT cells. Regulon analyses measure the change in transcript expression of all targets of a given transcription factor to understand the activity of that transcription factor in the cell. High SOX4 regulon expression in C1 and C7 is consistent with previous findings in mice showing that *SOX4* is important for early iNKT cell development^37^ (Figure 3A). The YY1 regulon, essential for Th2 cell differentiation,^38,39^ exhibited increased activity in C0, further suggesting some Th2-like features of this transitional population. Another regulon highly expressed in C0 was ATF3, which negatively regulates IFNγ expression in mouse NK cells,^40^ suggesting that this expression is restrained until cells have differentiated into Th1/17/NK-like. Interestingly, *CD79A* is a target of the ATF3, FOS, FOSL2, and JUND regulons, all with increased activity in C0. Correspondingly, the *CD79A* transcript was highly expressed in C0 (Figure 1F; C0: 3.34 avg log2FC), similar to a recent report on the transcriptome of human PB iNKT cells.^41^ However, *CD79A* encodes a B cell receptor co-factor protein,^42,43^ which we were unable to detect by flow cytometry (Figures S3B and S3C). Interestingly, the FOSL2 regulon, with high activity in C0 and C6, contains *MYBL1*, which has been associated with innateness in lymphocytes^44^ and is highly expressed in C2 and C5 (Figure 1F; Table S3), suggesting a sequential activation of transcription factors. The eomesodermin (EOMES) regulon, with high activity in C2 and C5, contains 33 target genes, including *GZMK*, *NKG7*, *KLRG1*, *CCL4*, and *LAG3*. EOMES (the protein encoded by *Eomes*) has been shown to regulate iNKT cell differentiation into iNKT1 cells and KLRG1-expressing iNKT cells in mice.^45^ Collectively, these transcriptomic data reveal similarities in mouse and human iNKT cell transcription factor gene expression while highlighting unique transcription factors important in driving human iNKT cell transcriptional diversity.

To further evaluate the differentiation trajectory of human iNKT cells, we performed cellular trajectory reconstruction analysis using gene counts and expression (CytoTRACE) and pseudotime trajectory analysis (Monocle3) (Figure 3B). CytoTRACE predicts the cellular differentiation state from an scRNA-seq-based determinant of developmental potential, assuming transcriptional diversity decreases throughout differentiation. In a complementary fashion, pseudotime infers the order of cells along a trajectory or related lineage. CytoTRACE analysis revealed that C1 and C7 represented the least differentiated clusters, correlating with their primarily thymic origin, which served as the root nodes for pseudotime analysis. Differentiation progressed through clusters C0 and C3 to the Th1/17/NK-like clusters (C2 and C5) in both algorithms, further supporting the transitional nature of C0 and C3.

Next, we evaluated regulons that correlated with the differentiation state. Regulons changing across pseudotime were defined for two trajectories (trajectory 1: C0, C1, C4, C6, C7 and trajectory 2: C0, C2, C3, C5) (Figures 3B, 3C, and S4A–S4D) or the entire pseudotime trajectory (trajectory 3; Figures S4E and S4F). Along trajectory 1, as cells differentiated from the thymus, BCL3 regulon activity increased between the precursor clusters and the transitional clusters (r = 0.77, Pearson correlation; Figures 3B–3D). Bcl3 is a pro-survival factor and stabilizes nuclear factor κB (NF-κB) p50 homodimers, promoting immunologic tolerance in mice.^46,47^ Accordingly, we saw high NFKB1 regulon activity (*NFKB1* encodes NF-κB p105, the p50 precursor) in C0, although NFKB2 and REL regulon activity was also increased (Figure 3C). The FOSL2 regulon also increased in activity along trajectory 1 (Figures 3B–3D). Several regulons exhibited increased activity along trajectory 1 and reduced activity along trajectory 2, including KLF transcription factor family members (KLF6, KLF10, and KLF16), along with ATF4 and ELF1. The Ets transcription factor ELF1 is required for the development and function of iNKT cells,^48^ while KLF10 restrains Th17-like differentiation in another innate lymphocyte population.^49^ Cellular development along trajectory 2 toward the Th1/17/NK-like clusters showed increasing EOMES regulon activity, among others (Figures 3B–3D). Finally, SOX4, important in early iNKT cell development in mice,^37^ had the strongest reduction in activity across pseudotime considering all cells (trajectory 3, r = −0.56, Pearson; Figures 3D, S4E, and S4F), suggesting less of a role as iNKT cells proceed through differentiation. Taken together, these findings begin to elucidate the transcriptional regulatory networks that change as human iNKT cells differentiate.

### Expression patterns reveal unique T_EM_ CD45RA^+^-like iNKT cells

**Figure 4.**
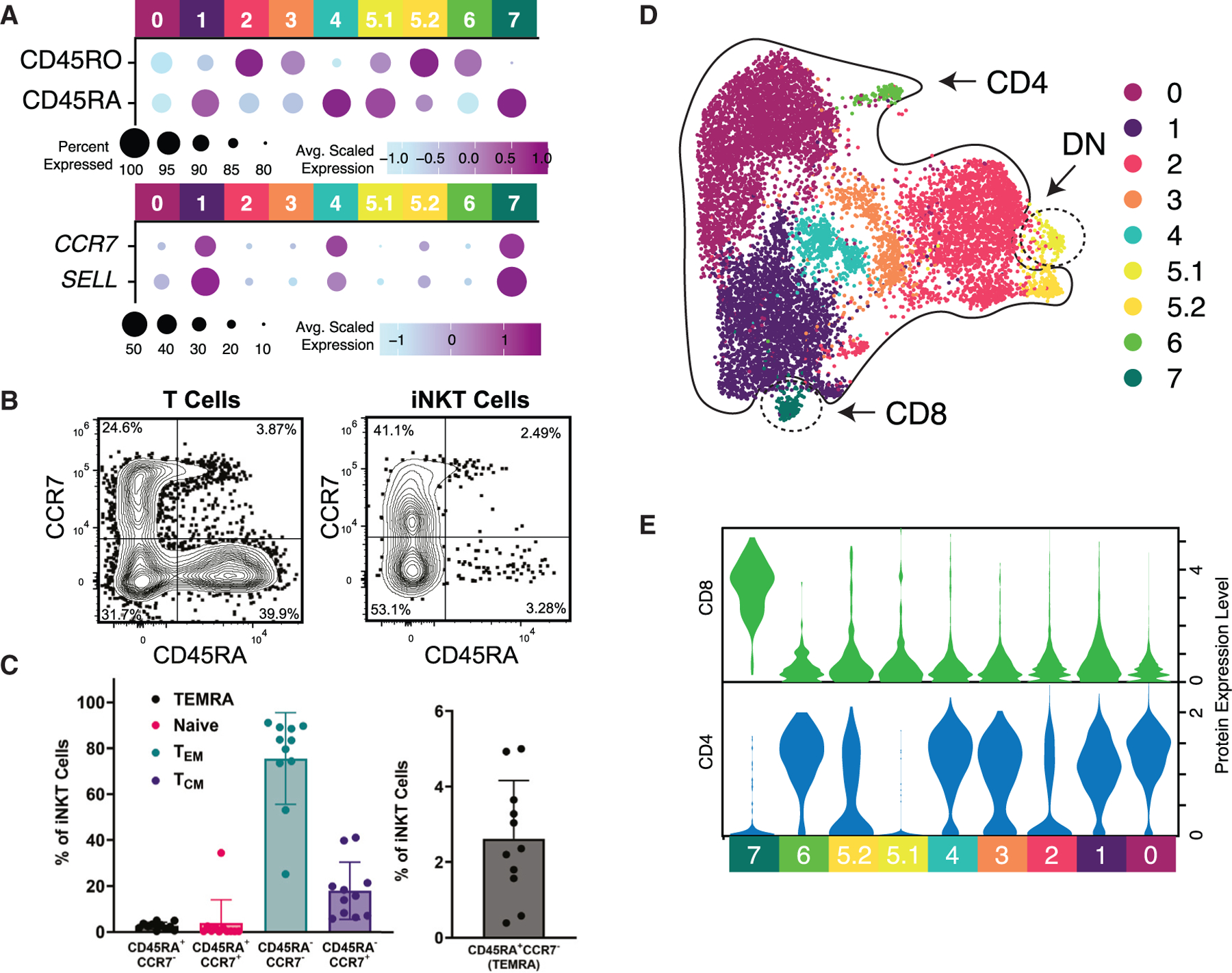
Gene and protein expression patterns demonstrate CD4^−^CD8^−^ T effector memory CD45RA^+^-like iNKT cells (A) Dot plot of CD45RO and CD45RA protein expression as measured by oligomer-conjugated antibodies and *CCR7* and *SELL* gene expression. For each image, the size of dot indicates the percentage of cells expressing the protein/gene of interest, and the color indicates the average scaled expression. (B) Representative flow cytometry dot plot showing expression of CD45RA and CCR7 for PB T cells (left) and iNKT cells (right). (C) Summary flow data of CD45RA and CCR7 expression revealing percentages of iNKT cells resembling T effector memory RA^+^ (TEMRA), naive, T effector memory (T_EM_), and T central memory (TCM) cells (left) and TEMRA cells (right, scale modified to enhance visibility). Bar graphs represent the mean ± SD, with individual donor data points plotted. (D) UMAP of iNKT cells colored by cluster assignment. Regions of UMAP are circled based on the observed protein expression of CD4 and CD8. (E) Violin plot of CD4 and CD8 protein expression separated by cluster. See also Figures S5 and S6.

The inclusion of oligomer-conjugated antibodies to detect CD45RA and CD45RO, together with mRNA expression of *CCR7* and *SELL* (gene encoding CD62L), allowed for the evaluation of naive and memory phenotypes of human iNKT cells, following the canonical definition in human conventional T cells (Figure 4A). Precursor clusters (C1, C4, and C7) had high expression of *CCR7* and *SELL*, with relatively increased CD45RA, similar to naive T cells and suggesting a naive/precursor signature (Figure 4A). These findings correlate with our CytoTRACE and pseudotime analyses and are further supported by the relatively increased expression of genes important for early iNKT cell development, including *LEF1*, *TOX2*, *SOX4*, and *ITM2A* (Figure 1F).^21,35,37,50,51^ Indeed, lymphoid enhancer-binding factor 1 (LEF1) expression appears to regulate CD62L expression in *ex*-*vivo*-expanded iNKT cells, albeit with a central memory phenotype possibly reflecting changes occurring during culture.^52,53^ Most remaining iNKT cells in our analysis displayed a T_EM_ signature (CD45RA^−^ with no/low *CCR7* expression) (Figure 4A). Surprisingly, a subset of cells within C5 (C5.1) predominantly expressed CD45RA with an absent *CCR7* transcript (Figure 4A), similar to TEMRA cells, conventional memory T cells with re-expression of CD45RA.^54^ Further, a published gene signature associated with conventional TEMRA cells was highly expressed in C5.1 (Figures S5A and S5B).^55^ We also detected these cells in PB by flow cytometry (Figures 4B, 4C, and S5C), although they represent a very small percentage of iNKT cells.

### CD4 and CD8 expression reveals a naive/precursor transcriptional profile of CD8^+^ iNKT cells and demonstrates that CD4 expression poorly delineates functional propensity

Due to the high dropout rate of *CD4* in scRNA-seq analyses, we utilized oligomer-conjugated antibodies to analyze CD4 and CD8 protein expression in addition to RNA expression (Figures 4D, 4E, and S6A). While CD8^+^ human iNKT cells have been previously demonstrated by flow cytometry,^31^ little is known about their function. Surprisingly, we found that CD8^+^ iNKT cells (C7) are characterized by a naive/precursor transcriptional profile (Figures 4A, 4D, and 4E). Most clusters expressed CD4, while C5.1 was double negative (DN; CD4^−^CD8^−^) (Figures 4D and 4E). Th2-like mouse iNKT cells have been shown to express CD4, which is also expressed on a subset of Th1-like iNKT cells.^2,20,34^ Similarly, C2 represents a human CD4^+^ Th1/17/NK-like population. This cluster had high expression of *TBX21*, *RORC*, and many cytotoxicity and NK genes. These findings suggest that CD4 may not best distinguish functionally distinct subsets of iNKT cells.

### Transcriptional analysis of human PB-derived iNKT cells reveals further heterogeneity, particularly in the CD8^+^ population

**Figure 5.**
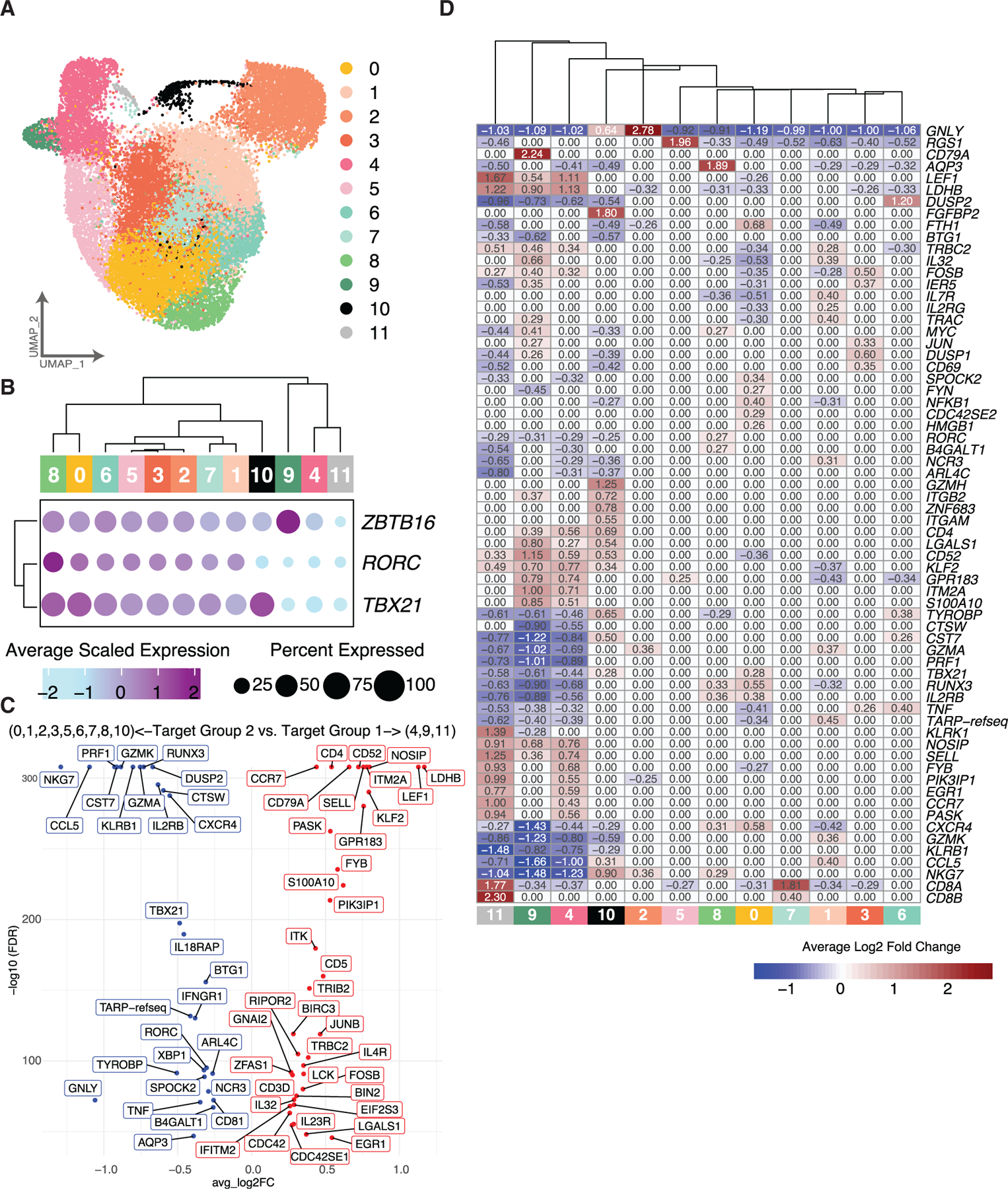
Transcriptional heterogeneity of human PB-derived iNKT cells (A) UMAP of all iNKT cells isolated from PB donors. Cells are colored by cluster assignment. (B) Expression dot plots of key transcription factors. Size of dot indicates the percentage of cells expressing the indicated gene, and the color indicates the average scaled expression. (C) Volcano plot of top differentially expressed genes separating PB-C4, PB-C9, and PB-C11 from all other clusters. Each dot is labeled by the gene name, and the color of the dot and label indicates increased expression based on each of the target groups (red: upregulated in target group 1, blue: upregulated in target group 2). (D) Heatmap of the top 10 differentially expressed genes identified by each cluster. Each cell is colored by the average log2FC of each gene identified in each cluster. A value of 0 indicates the gene was not identified as having significantly increased or decreased expression for the labeled cluster. See also Figures S5 and S6 and Table S4.

**Figure 6.**
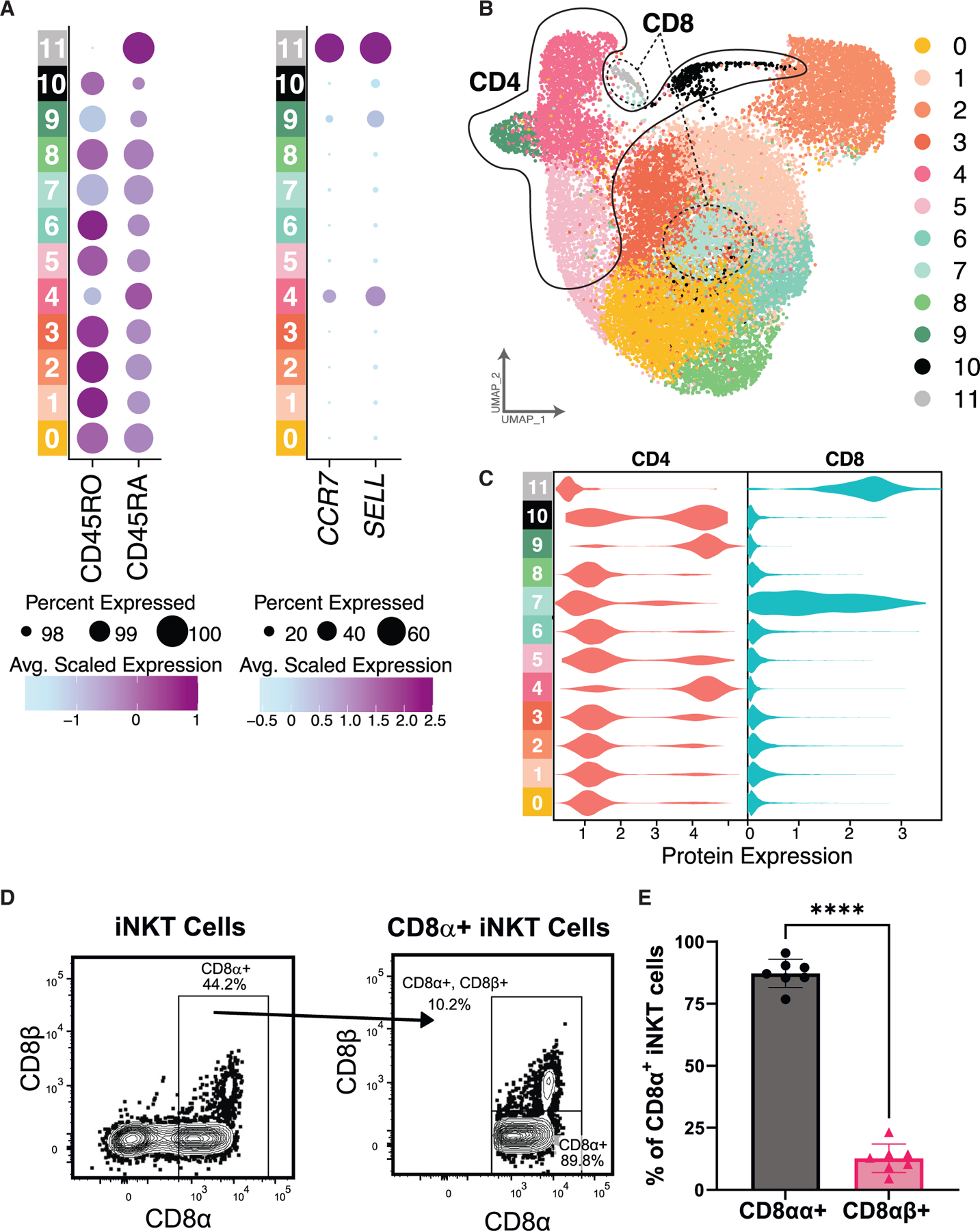
Gene and protein expression patterns of human PB iNKT cells facilitate detection of naive/precursor and Th1/17/NK-like CD8^+^ iNKT cells (A) Dot plot of CD45RO and CD45RA protein expression as measured by oligomer-conjugated antibodies and *CCR7* and *SELL* gene expression. For each figure, the size of dot indicates the percentage of cells expressing the protein/gene of interest, and the color indicates the average scaled expression. (B) UMAP of iNKT cells colored by cluster assignment. Regions of UMAP are circled based on the observed protein expression of CD4 and CD8. (C) Violin plot of CD4 and CD8 protein expression separated by cluster. (D) Representative flow cytometry dot plot using antibodies to CD8α and CD8β. (E) Summary flow data (*n* = 8) of CD8α and CD8β expression in CD8α^+^ iNKT cells revealing percentages of iNKT cells expressing CD8αα homodimer (no CD8β detection) or CD8αβ heterodimer (co-expression of CD8α and CD8β). Bar graphs represent the mean ± SD, with individual donor data points plotted. ****p < 0.0001 by paired t test. Data were pooled from at least three independent experiments. See also Figure S7.

To study PB-derived iNKT cell heterogeneity more robustly, we utilized a custom targeted scRNA-seq panel with 435 genes on 24,758 cells from 10 donors (Figures 5A and S6B; Table S2). The overall correlation between the top variable genes in the WTA panel and the targeted panel was very high (r = 0.9, *p* < 2.2e–16, Pearson correlation) and revealed a striking similarity in the expression pattern of key genes despite having an overall smaller gene space (Figures S6C and S6D). Similar to our previous analysis, we identified naive/precursor, transitional, and Th1/17/NK-like clusters within human PB iNKT cells (designated PB-C#) based on the differential expression of transcription factors, cytotoxicity genes, cytokine receptors, and naive/memory signatures (Figures 5B–5D, 6A, and S7A; Table S4). Th1/17/NK-like clusters (PB-C0–3, PB-C5–8, and PB-C10) co-expressed *TBX21* and *RORC*, along with the highest levels of cytotoxicity genes, as compared to the transitional cluster (PB-C9) and naive/precursor clusters (PB-C4 and PB-C11) (Figures 5B–5D and S7A). Clusters with high expression of cytotoxicity genes were similarly seen in a previously published transcriptional analysis of human PB iNKT cells,^41^ though with high differential expression of *GZMB*, which was not among our top differentially expressed genes (instead included *GZMA* and *GZMK*). This corroborates another report showing a lack of granzyme B usage in human iNKT cells.^26^ Naive/precursor and transitional clusters were characterized by low/absent expression of these cytotoxicity genes and high expression of genes characteristic of naive and Th2 T cells, including *SELL*, *LEF1*, *ITK*, *CD52*, *CD4*, and *IL4R* (Figures 5C and 5D), similar to prior results.^41^ Interestingly, *ITK* has been shown to provide important signals for the differentiation of mouse iNKT cells.^56^ The cluster with the highest *RORC* expression (PB-C8) also had exclusive high expression of *AQP3* (Figure 5D; 1.89 avg log2FC), as previously demonstrated specifically in iNKT17 cells in mice.^57^

Four clusters express CD4 (PB-C4, PB-C5, PB-C9, and PB-C10) (Figures 6B and 6C). PB-C9 also had the highest expression of *ZBTB16*, while PB-C5 and PB-C10 lie among the Th1/17/NK-like clusters (Figure 5B). PB-C10 highly expressed *GZMH* and *FGFBP2* (Figure 5D), the protein product of which is secreted by cytotoxic T and NK cells^58^ (Figures 5D and S7A). Interestingly, we found two CD8^+^ iNKT clusters with distinct transcriptional patterns—one within the naive/precursor group of clusters (PB-C11), similar to data from our multi-tissue experiment, and one in the Th1/17/NK-like group of clusters (PB-C7) (Figures 5B, 5D, and 6A–6C). PB-C11 had increased expression of both *CD8A* and *CD8B*, while PB-C7 had much higher *CD8A* expression relative to *CD8B* (Figure 5D; PB-C11, *CD8A*: 1.77 and *CD8B*: 2.30 avg log2FC; PB-C7, *CD8A*: 1.81 and *CD8B*: 0.40 avg log2FC), suggesting that iNKT cells in PB-C7 may express CD8αα homodimers, similar to highly cytotoxic NK cells and other innate lymphocytes.^59–61^ Validation by flow cytometry showed 87% of PB-derived CD8^+^ iNKT cells stained only with antibody detecting the CD8α monomer and not with antibody detecting the CD8β monomer, indicating expression of the CD8αα homodimer (Figures 6D, 6E, and S7B). Taken together, these data reveal heterogeneity within CD4^+^ and CD8^+^ human iNKT cell populations and suggest these markers may not be ideal correlates of iNKT cell function.

### Distinct biological processes in iNKT cells differ by hematologic tissue

**Figure 7.**
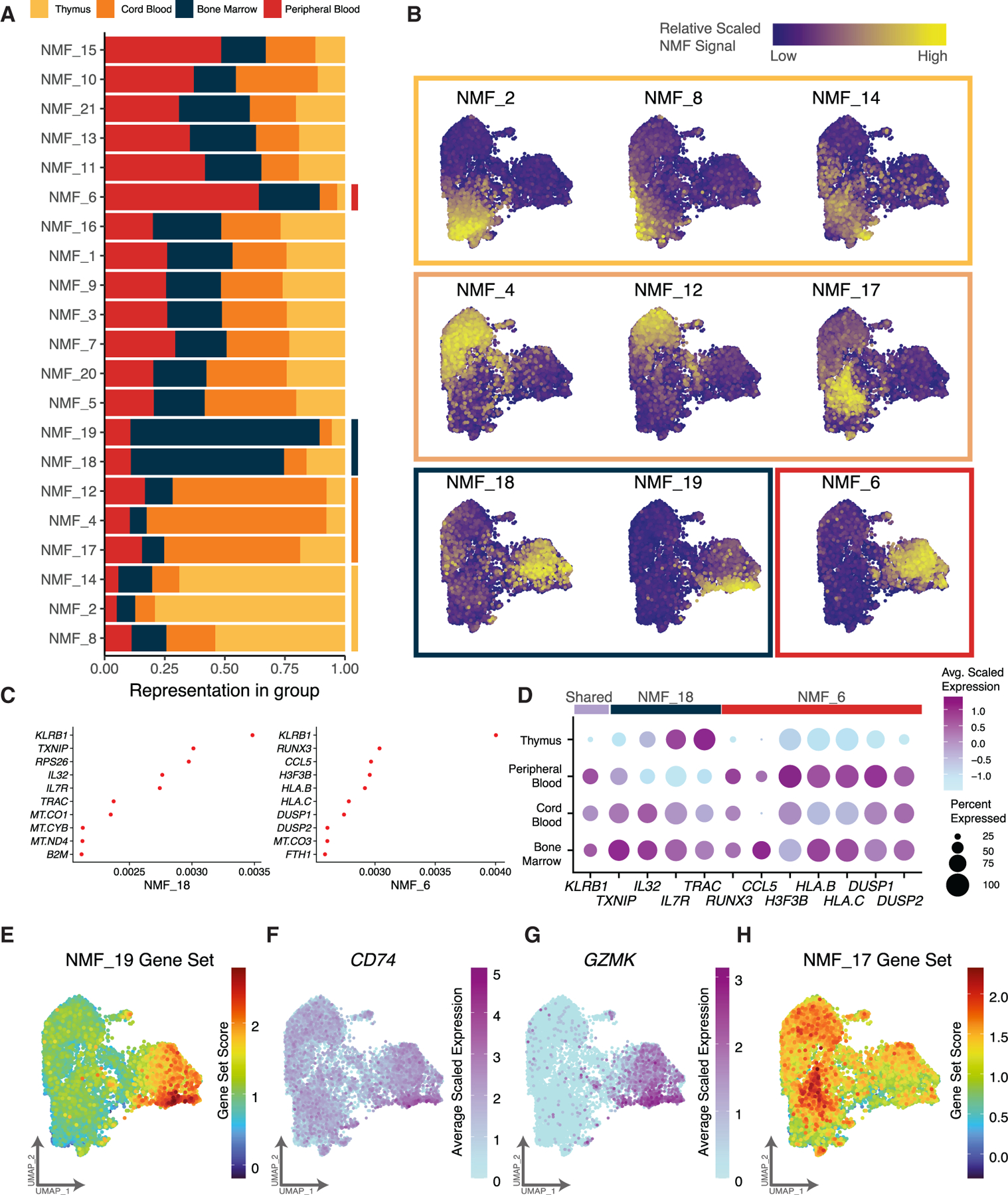
Transcriptional heterogeneity of human iNKT cells from different tissue sources within and between clusters (A) Bar plot highlighting differing proportions of non-negative matrix factorization (NMF) factors identified across each tissue type. Proportion of cells belonging to each factor (or biological process) is indicated by color. (B) UMAP representation of iNKT cells colored by scaled NMF signal of 9 NMF factors that are highly represented by each tissue type. Each UMAP is grouped by a square outline that is colored by the tissue type showing the strongest contribution. (C) Visual loadings of the top genes contributing to the two indicated NMF factors. (D) RNA expression of a subset of key genes for two NMF factors separated by tissue type. The size of the dot indicates the percentage of cells expressing the indicated gene, and the color indicates the average scaled expression. (E) Gene set expression of genes driving NMF_19 overlaying the iNKT UMAP. Each cell is colored by the scaled module score of the NMF_19 gene set. (F and G) UMAP of iNKT cells colored by expression of annotated gene. (H) Gene set expression of genes driving NMF_17 overlaying the iNKT UMAP. Each cell is colored by the scaled module score of the NMF_17 gene set.

Although we found that PB-derived iNKT cells recapitulated the clusters observed in the multi-tissue experiment, we sought to further evaluate transcriptional differences between the hematologic tissues. We utilized non-negative matrix factorization (NMF) to evaluate biological processes differentially represented in each tissue type. We found several NMF factors (2, 4, 6, 8, 12, 14, 18, and 19) differentially represented between tissues, while others were relatively evenly distributed (Figures 1C, 7A, and 7B). NMF_6 (PB) and NMF_18 (BM) occupy the same clusters (C2 and C5) yet contain different genes driving each biological process. While *KLRB1* is the primary gene associated with both factors, NMF_6 is strongly driven by *RUNX3*, *CCL5*, *H3F3B*, and *HLA.B*, while NMF_18 is associated with *TXNIP*, *IL32*, *TRAC*, and *IL7R* (Figures 7C and 7D), suggesting that while most features of the Th1/17/NK-like clusters are shared by PB- and BM-derived iNKT cells, they contain underlying biological gene programs that distinguish them. NMF_19 (containing *CCL5*, *CD74*, *GZMK*, *CXCR4*, *CST7*, and *NKG7*) is dominated by BM-derived cells (Figure 7A) and clearly highlights the lower region of the uniform manifold approximation and projection (UMAP) spanning C2 and C5, aligning with the pseudotime trajectory (Figures 1D, 3B, and 7E–7G). NMF_17 (containing *RGS1*, *CXCR4*, *CD69*, *BTG1*, *JUN*, and *KLF6*) overlays the pseudotime trajectory between naive/precursor clusters C1 (largely thymic derived) and C4 (thymic and PB) (Figures 3B, 7B, and 7H). The CXCR4-CXCL12 signaling axis (also containing Jun) is critical for cell development and migration in lymphocytes,^62,63^ and mice with CXCR4 deficiencies have decreased functional NK cells in various tissues,^64^ suggesting that NMF_17 represents early transcriptional changes driving iNKT cell maturation and thymic egress. By integrating iNKT cells across tissue types and evaluating shared regulatory processes, these data begin to elucidate the underlying transcriptional mechanisms driving iNKT cell development and cellular diversity.

## DISCUSSION

Evolutionarily conserved iNKT cells are a critical component of immune responses in a variety of diseases and a novel approach to universal donor cellular therapies.^65–67^ A better understanding of human iNKT cell biology is required to realize their full potential. Herein, we report an integrated transcriptional analysis of human iNKT cells from PB, CB, thymus, and BM, revealing critical insights into their heterogeneity in these tissues.

Our data demonstrate that human iNKT cells form a differentiation trajectory, which, when limited to CD24^−^ iNKT cells, nevertheless appears to originate from an early precursor stage, characterized by the expression of genes similarly important in mouse iNKT cell development, including *LEF1*, *TOX2*, *SOX4*, and *ITM2A*.^35,37,50,51,68^ *Sox4* induces microRNA-181 to enhance TCR signaling in iNKT precursors,^37^ while ITM2A expression correlates with positive selection of conventional T cells and is expressed in ST0 iNKT cells.^35,68,69^ *Lef1* is expressed in ST0 and early differentiating iNKT cells and has been implicated in iNKT2 differentiation in mice.^21,35,51^ These human precursor iNKT cells also expressed *CCR7*, associated with an immature phenotype of iNKT cells in mice,^70^ and CD45RA, corresponding with human naive conventional T cells (CD45RA^+^CCR7^+^). These cells also align with ST0 mouse iNKT cells when integrated with a recently published mouse dataset.^25^ Most of the remaining iNKT cells in our study express a T_EM_ profile (CD45RA^−^CCR7^−^), while a small population of DN iNKT cells display a TEMRA-like expression pattern (CD45RA^+^CCR7^−^). Among conventional T cells, TEMRA cells are mostly CD8^+^, with the highest IFNγ production and cytotoxic function, and increase in PB and BM with age.^71–73^ Rare CD4^+^ TEMRA cells also demonstrate cytotoxic function.^74^ Thus, TEMRA iNKT cells share many similarities with TEMRA conventional T cells. Our analysis has also started to disentangle the complex network of transcription factors acting in concert to alter the differentiation state of human iNKT cells and highlights several regulons that change over the course of differentiation.

Mouse iNKT cells can emerge from the thymus at multiple points along the differentiation trajectory.^21,68^ Accordingly, we found that the naive/precursor clusters were populated by both thymic-derived and PB-derived iNKT cells. The transitional cluster C0 encompassed a mix of cells displaying naive and memory signatures (even among the exclusively CB-derived cells). These data echo earlier observations in CB-derived conventional T cells^75^ yet differ from the purely naive phenotype observed in CB MAIT cells.^76^ Our differentiation trajectory correspondingly suggests that these cells represent a transitional phenotype between naive/precursor iNKT cells and Th1-like iNKT cells, similar to recent findings in mice.^21,22^ In these studies, some mouse iNKT2 cells represented a transitional population with iNKT1 and iNKT17 cells independently branching off (while other iNKT2 cells appeared more terminally differentiated). Our human data similarly suggest some potential Th2-like features of this transitional population, such as increased IL-4R expression and YY1 regulon expression, though *IL17RB* expression was absent, potentially reflecting species differences or the lack of a true iNKT2 population in human iNKT cells. In contrast to the distinct mouse iNKT1 and iNKT17 populations, our study revealed the co-expression of *TBX21* and *RORC* in some human iNKT cells, suggesting a combined Th1/17 signature as has been described for human MAIT cells^24,77^ and indeed aligning with them in our integrated datasets. Some cells also had an exclusive Th1 signature, though no exclusively Th17-like iNKT cells were detected. The Th1/17/NK-like population was transcriptionally characterized by many features of other cytotoxic innate lymphocytes, including cytokine receptors expressed by NK cells^78^ and granzyme K.^26^

While many studies of iNKT cells compare CD4^+^ to CD4^−^CD8^−^ (DN) cells, CD8^+^ iNKT cells are a relatively understudied population, likely owing to their rarity in humans and absence in mice,^31,79,80^ though this has been called into question.^81^ Our data demonstrate a CD8^+^ cluster with a naive/precursor profile in both of our experiments, as well as a CD8^+^ cluster with a Th1/17/NK-like transcriptional profile in our PB-only experiment, which may be related to substantially different numbers of iNKT cells analyzed between the two experiments. We further showed that the majority of human PB iNKT cells express CD8αα monomers, similar to other innate lymphocyte populations.^61,82^ Analogous to previous findings in mice that iNKT1 cells can be CD4^+^ or CD4^−^,^20,34^ we found CD4^+^ cells within the Th1/17/NK-like clusters. Our data suggest that the presence or absence of CD4 and CD8 expression does not clearly delineate functionally distinct subsets.

Similar to a recently published transcriptional analysis of PB-derived iNKT cells, we show that iNKT cells with high expression of cytotoxicity genes and chemokines cluster distinctly from iNKT cells with high expression of genes important in naive/precursor cells, such as *SELL* and *LEF1*.^41^ Another transcriptomic study of several innate lymphocyte populations from human PB and thymus suggested the presence of iNKT cells with a combined Th1/17 transcriptional signature, a TEMRA-like gene signature, and a CD8αα-like gene signature in their transcriptional analysis.^83^ They did not, however, detect a *CD4*-expressing population with a Th1/17/NK-like signature. Our inclusion of oligomer-conjugated antibodies to CD45RO, CD45RA, CD4, and CD8 and further flow cytometry validation allowed for definitive detection of iNKT cells with co-expression of Tbet and RORγt, TEMRA-like iNKT cells (CD45RA^+^CCR7^−^), CD8αα^+^ iNKT cells, and CD4^+^ iNKT cells with a Th1/17/NK-like gene signature. Further, we highlight unique gene signatures in CB-derived and BM-derived iNKT cells, even when compared to PB-derived iNKT cells within the same clusters.

Altogether, our results reveal critical insights into the diversity of human iNKT cells and highlight important similarities and differences between human and mouse iNKT cells. Key differences may manifest in multiple ways. For example, mouse iNKT2 and iNKT17 cells can suppress acute GVHD through the regulation of proinflammatory conventional T cells, while cytotoxic iNKT1 cells cannot.^18^ Conversely, in xenograft GVHD models, human iNKT cells with cytotoxic capacity (at least partly corresponding to our iNKT1/17/NK-like clusters) suppress GVHD through the killing of antigen-presenting cells,^84^ with correlative human GVHD analyses suggesting similar results.^13,85^ Whether species-specific patterns of distinct iNKT cell subsets impact functional outcomes remains to be determined. Further, the potential manifestation of these differences in other disease settings warrants further investigation. IL-17RB is a specific component of the receptor for IL-17E, also known as IL-25, a major Th2 response mediator.^86^ iNKT cells have been shown to play a pathologic role in asthma and airway diseases in mouse models,^87–89^ and the impact of the lack of *IL17RB* expression in human iNKT cells on such diseases remains unknown.

Defining the transcriptome of human iNKT cells from hematologic tissues in homeostasis provides a resource for future experimentation to demonstrate differential function and understand changes in various disease states, in different immune contexts, and during *ex vivo* expansion. Determining how these transcriptionally distinct cells may be differentially activated by various glycolipids and the potential for tissue-specific patterns of activation remains another important goal. These data lay the groundwork for optimized development of iNKT-based cellular therapies and provide a critical resource toward realizing that translational goal.

### Limitations of the study

Our work defines the transcriptomic heterogeneity of human iNKT cells from multiple hematologic tissues and provides a valuable resource for further study of these cells, yet there are several limitations to this work. First, the challenges associated with procuring healthy human tissues precluded our ability to analyze matched samples, particularly for all included tissues, and we chose not to match only some of the tissues. As such, we cannot exclude a donor-dependent effect of the results. Additionally, as this work was conducted as secondary research on de-identified samples without data on the sex of the donors, we cannot determine the impact of sex as a biological variable on our results. As our tissues of origin also reflect distinct time points across the age spectrum, we cannot exclude an age-dependent influence on our findings. Indeed, multiple mouse and human studies have suggested changes to iNKT cells with aging, progressing from a Th2-like iNKT cell predominance early in life to a greater relative abundance of Th1-like iNKT cells over time.^32,33,90,91^ However, it remains important to note that all described clusters were found in adult PB samples. Also, our work specifically focuses on type I NKT (iNKT) cells, and the sorting strategy used precludes analysis of type II NKT cells and rare atypical type I NKT cells with alternate TCRs.^92^ A final limitation of this work includes the need for validation of these transcriptional signatures and their functional implications for the abundant iNKT cell populations in the tissues analyzed. We are hopeful that this resource will provide the necessary foundation for future functional studies to assess the effector functions of iNKT cells with these distinct signatures, including proliferative capacity, cytokine production, cytotoxic function, and immunosuppressive ability, particularly in response to specific stimuli.

## RESOURCE AVAILABILITY

### Lead contact

Requests for further information and resources should be directed to and will be fulfilled by the lead contact, Melissa Mavers (mmmavers@wustl.edu).

### Materials availability

This study did not generate new unique reagents.

### Data and code availability

scRNA-seq data have been deposited in the NCBI Gene Expression Omnibus at GEO: GSE261557 and are publicly available as of the date of publication. All original code has been deposited at GitHub at https://doi.org/10.5281/zenodo.14984003 and is publicly available. For external sample integration, data were downloaded from the associated repositories for Bugaut et al.^24^ and Wang et al.^25^ Any additional information required to reanalyze the data reported in this paper is available from the lead contact upon request.

## STAR★METHODS

### EXPERIMENTAL MODEL AND STUDY PARTICIPANT DETAILS

#### Human sample acquisition

This study was determined by the Stanford University Institutional Review Board to be IRB-exempt as secondary research on deidentified samples originally acquired through IRB-approved research protocols with participant (or parental) consent. Thymic tissue (n=3) was acquired from pediatric patients with congenital cardiac disease undergoing surgical repair (excluding those with Chromosome 21q11.2 Deletion (DiGeorge) syndrome or other known immune disorders). Bone Marrow (n=2) was acquired from healthy adult donors for bone marrow transplantion. Peripheral blood samples (n=3 for multi-tissue experiment and n=10 for peripheral blood-only experiment) were obtained from adult donors of platelets (leukoreduction system chambers). Cord blood (n=3) was collected from donors at the time of parturition.

### METHOD DETAILS

#### Thymus samples

Thymic tissue was mechanically dissociated in a petri dish on ice in cold PBS with 10% FBS (Gibco, #26–140-079) and 100mg/L DNase I (Sigma-Aldrich, #10104159001) and passed through a cell strainer. Mononuclear cells were collected following Ficoll-Paque density gradient centrifugation (Cytiva, #17144003). Cells were then cryopreserved in 50% XVivo10 (Lonza #04–380Q) supplemented with 5% human AB serum (Sigma-Aldrich, #H4522) and 50% Cryoprotective freezing media (Lonza #12–132A). Samples were thawed in PBS (containing calcium and magnesium) supplemented with 3% FCS and 100 mg/L DNase I prior to iNKT cell isolation.

#### Peripheral blood samples

Peripheral blood mononuclear cells were isolated from leukoreduction system chambers using Ficoll-Paque density gradient centrifugation (Cytiva, #17144003). For peripheral blood-only experiment, cells were then subject to iNKT cell enrichment as outlined below. For multi-tissue experiment, cells were cryopreserved in 50% XVivo10 (supplemented with 5% human AB serum) and 50% Cryoprotective freezing media and thawed in PBS (containing calcium and magnesium) supplemented with 3% FCS and 100mg/L DNase I prior to iNKT cell enrichment (as above).

#### Cord blood samples

Mononuclear cells were prepared from cord blood samples by Ficoll-Paque density gradient centrifugation and CD34^+^ cells removed (Miltenyi Biotec #130–100-453). Remaining cells were cryopreserved in 50% XVivo10 (supplemented with 5% human AB serum) and 50% Cryoprotective freezing media and thawed in PBS (containing calcium and magnesium) supplemented with 3% FCS and 100mg/L DNase I prior to iNKT cell enrichment (as above).

#### Bone marrow samples

Bone marrow specimens were collected from clinically prepared bone marrow harvest bags. Specimens were visually inspected and mechanically dissociated when necessary, then passed through a cell strainer. Mononuclear cells were collected using Ficoll Paque density gradient centrifugation, cryopreserved in 50% XVivo10 (supplemented with 5% human AB serum) and 50% Cryoprotective freezing media, and thawed in PBS (containing calcium and magnesium) supplemented with 3% FCS and 100mg/L DNase I prior to iNKT cell enrichment (as above).

#### Magnetic bead enrichment of iNKT cells and fluorescence-activated cell sorting

For scRNA-seq analyses, human iNKT cells were enriched by magnetic bead-based positive selection (EasySep^™^ Release Human PE Positive Selection Kit, Stem Cell Technologies, #17654) utilizing PE-conjugated anti-Vβ11 antibody (C21, Beckman Coulter, #IM2290), followed by bead release per manufacturer instructions. Cells then underwent simultaneous additional staining with fluorochrome-conjugated antibodies, Sample Tag antibodies (BD Biosciences, #633781), and oligomer-conjugated antibodies. Samples were individually subjected to fluorescence activated cell sorting (FACSAria II, 4-way purity setting) for selection of live(DAPI^-^) singlet CD19^−^CD14^−^CD24^−^CD3^+^Vα24^+^Vβ11^+^ cells. Fluorochrome-conjugated antibodies included: APC-Cy7 CD19 (SJ25C1, BD Biosciences, #557791), APC-H7 CD14 (MØP9, BD Biosciences, #560180), APC-Cy7 CD24 (ML5, Biolegend, #311132), PerCP-Cy5.5 CD3 (UCHT1, Biolegend, #300430), FITC Vα24 (C15, Beckman Coulter, #IM1589), and PE Vβ11 (C21, Beckman Coulter, #IM2290). Oligomer-conjugated antibodies included Oligo-CD4 (SK3, BD AbSeq, #940001), Oligo-CD8 (RPA-T8, BD AbSeq, #940003), Oligo-CD45RA (HI100, BD AbSeq, #940011), and Oligo-CD45RO (UCHL1, BD AbSeq, #940022). Cell counts were roughly normalized between tagged samples and pooled prior to library preparation.

#### Flow cytometry analyses

For flow cytometry analyses, iNKT cells were enriched from human PB (LRS chambers) or CB using magnetic bead-based positive selection (Miltenyi Biotec, anti-iNKT microbeads, #130–094-842). Unstimulated single cell suspensions were stained with FC blocking reagent (Miltenyi, #130–059-901) for 15 minutes at 4°C. Then a combination of the following antibodies was added and stained for 30 minutes at 4°C in the dark: BUV395 CD3 (OKT3, Biolegend, #317308), BV421 CD3 (OKT3, Biolegend, #300433) *[TEMRA panel only]*, BUV496 CD8α (RPA-T8, BD, #612942), BV785 CD4 (OKT4, Biolegend, #317441), FITC Vα24 (6B11, Biolegend, #342905 ), APC Vβ11 (C21, Beckman, #A66905), PE Vβ11 (C21, Beckman, #IM2290) *[TEMRA and Transcription Factor panels only]*, AF700 CD19 (SJ25C1, Biolegend, #363034), PE-Cy7 CD19 (HIB19, Biolegend, #302215) *[CD79a panel only],* AF700 CD14 (M5E2, Biolegend, #301822), BUV395 CD45RA (HI100, Biolegend, #304190) *[TEMRA Panel only]*, PE-Cy7 CCR7 (2-L1-A, BD, #567314) *[TEMRA Panel only]*, APC CD45RO (UCHL1, Biolegend, #304210) *[TEMRA Panel only]*, BV421 CD8β (2ST8.5H7, BD, #742390) *[CD8*αα *Panel only]*, PE CD8α (SK1, BD, #742390) *[CD8*αα *Panel only]*, and Fixable Viability Dye e780 (ThermoFisher, #65–0865-14). Samples were then washed in MACS buffer and fixed with 4% PFA [*TEMRA and CD8*αα *panels only*] prior to analysis. ***For CD79a panel***, unstimulated cells were fixed for 20 min with FoxP3 Fix/Perm kit (Invitrogen, #00–5523-00) and then stained intracellularly in 1X Perm wash buffer with Fc blocking reagent (Miltenyi, #130–059-901) for 15 minutes followed by BV421 CD79a (HM47, Biolegend, #333519) in 1X perm wash for 30min at 4°C in the dark prior to analysis. ***For transcription factor panel***, cells were stimulated by plating 5×10^5^ enriched iNKT cells and 5×10^6^ lethally irradiated PBMCs [10:1 (PBMC:iNKT)] per well in a 96-well round bottom plate in 200uL of Xvivo10 supplemented with 5% Human Ab serum and the following cocktails at 37°C incubator with 5.2% CO*2*: ***STIM A:*** IL-1β (10ng/mL, Biolegend, #579402), TGFβ (5ng/mL, Miltenyi, #130–095-066), IL-23 (20ng/mL, Biolegend, #574102), αGC (100ng/mL, AdipoGen, #AGCN20013M001), IL-2 (200IU/mL, NCI). ***STIM B:*** IL-1β (10ng/mL, Biolegend, #579402), TGFβ (5ng/mL, Miltenyi, #130–095-066), IL-6 (10ng/mL, Biolegend, # 570802), αGC (100ng/mL, AdipoGen, #AGCN20013M001), IL-2 (200IU/mL, NCI). ***STIM C:*** αGC (100ng/mL, AdipoGen, #AGCN20013M001), IL-2 (200IU/mL, NCI). ***No Stim (control):*** IL-2 (200IU/mL, NCI). After 72 hours, cells were harvested and single cell suspensions were stained with Fc blocking reagent (Miltenyi, #130–059-901) for 15min at 4°C. Then a combination of the following was added and stained for 30 minutes at 4°C in the dark: BUV395 CD3 (OKT3, Biolegend, #317308), BUV496 CD8α (RPA-T8, BD, #612942), BV785 CD4 (OKT4, Biolegend, #317441), FITC Vα24 (6B11, Biolegend, #342905 ), PE Vβ11 (C21, Beckman, #IM2290), AF700 CD19 (SJ25C1, Biolegend, #363034), AF700 CD14 (M5E2, Biolegend, #301822), and Fixable Viability Dye e780 (ThermoFisher, #65–0865-14). Samples were then washed in MACS buffer and fixed using the FoxP3 Fix/Perm kit (Invitrogen, #00–5523-00*)* for 1hr at 4°C in the dark. Cells were then stained intracellularly in 1X Perm wash buffer with Fc blocking reagent (Miltenyi, #130–059-901) for 15 minutes followed by a combination of the following antibodies overnight at 4°C in the dark: BV421 Tbet (4B10, Biolegend, #644832) and PE RORγt (Q21–559, BD, #563081) or ISOTYPE- BV421 (MOPC-21, Biolegend, #400157) and ISOTYPE-PE (MOPC-21, Catalog # 557872). All data was collected on a Cytek Aurora (SpectroFlow software), and analyses were performed using FlowJo. iNKT cells (Vα24^+^Vβ11^+^) were analyzed after doublet, viability, T cell (CD3^+^CD19^-^CD14^-^) discrimination.

#### Preparation and sequencing of scRNA-seq libraries

Cell capture and library preparation were completed using the BD Rhapsody mRNA and AbSeq reagent kit (BD Biosciences, #633774) following the manufacturer instructions. Briefly, cells were captured with beads in a microwell plate, followed by cell lysis, bead retrieval, cDNA synthesis, template switching and Klenow extension. scRNA-seq libraries were generated using the Rhapsody platform (Becton Dickenson) employing a targeted panel (PB only, Table S2) or targeted panel and whole transcriptome analysis (multi-tissue experiment), as well as for sample tags and AbSeq antibodies. Sequencing was performed on a NovaSeq6000 (Illumina). Data was processed using the BD Rhapsody pipeline (Seven Bridges) followed by analysis in R.

#### scRNA-seq data preprocessing for multi-tissue whole transcriptome data

Expression tables (RSEC_MolsPerCell.csv) and sample tags (Sample_Tag_Calls.csv) were downloaded from Seven Bridges and analyzed using Seurat (v4.3.0 and v5.0.3). First, separate assays were created using the CreateSeuratObject function and percent mitochondrial DNA, nCount_RNA and nFeature_RNA were evaluated for each cell. Cells were maintained for downstream processing if they met the following criteria: nFeature_RNA > 200; nFeature_RNA < 4000; nCount_RNA < 20000; percent.mito < 20. Cells with sample tags labeled ‘‘Undetermined’’ or ‘‘Multiplets’’ were filtered out. Each sample was scaled and normalized using Seurat’s ‘SCTransform’ function to correct for batch effects (with parameters: vars.to.regress = c(“nCount_RNA”), dims = 1:20). Any merged analysis or subsequent subsetting of cells/samples underwent the same scaling and normalization method. Cells were clustered using the original Louvain algorithm and top 10 PCA dimensions via ‘FindNeighbors’ and ‘FindClusters’ (with parameters: resolution = 0.5) functions. After initial subsetting and clustering, a clear population of infiltrating macrophages were identified exhibiting higher expression of *FCGR3A, LYN, FCR1G, IL1B*. The macrophage/antigen presenting cluster was filtered out and we subsequently used Harmony (group.by.vars = “Sample_Tag”, reduction = “pca”, assay.use = “SCT”) before rerunning RunUMAP, FindNeighbors and FindClusters to annotate final object (dims=1:20). The resulting merged and normalized matrix was used for the subsequent analysis. To evaluate cluster resolution we utilized clustree before settling on cluster assignment. ADT data was normalized using NormalizeData command with method = ‘‘CLR’’ and a margin of 2. Of note, previous studies have also required removal of a small subcluster of antigen presenting cells.^93^ All initial clustering was completed in Seurat version 4.3.0 and UpdateSeuratObject was used to update object for use in Seurat version 5.0.3 for subsequent analyses.

#### scRNA-seq data preprocessing for multi-tissue and peripheral blood-only targeted panel data

Expression tables (MolsPerCell.csv) and sample tags (Sample_Tag_Calls.csv) were downloaded from Seven Bridges and analyzed using Seurat (v4.3.0 and 5.0.3). RSEC_MolsPerCell file was used for the Multi-tissue targeted panel and DBEC_MolsPerCell used for the peripheral blood-only targeted panel. First, separate assays were created using the CreateSeuratObject function. Each sample was scaled and normalized using Seurat’s ‘SCTransform’ function to correct for batch effects (with parameters: vars.to.regress = c(“nCount_RNA”), dims = 1:20). Any merged analysis or subsequent subsetting of cells/samples underwent the same scaling and normalization method. Cells were clustered using the original Louvain algorithm and top 10 PCA dimensions via ‘FindNeighbors’ and ‘FindClusters’ (with parameters: resolution = 0.5) functions. After initial subsetting and clustering, a clear population of infiltrating macrophages were identified exhibiting higher expression of *FCGR3A, LYN, FCR1G, IL1B*. The macrophage/suspected antigen presenting cluster was filtered out along with cells with sample tags labeled ‘‘Undetermined’’ or ‘‘Multiplets’’. Only the Multi-Tissue sample subsequently used Harmony (group.by.vars = “Sample_Tag”, reduction = “pca”, assay.use = “SCT”), while both datasets underwent the same downstream preprocessing using RunUMAP, FindNeighbors and FindClusters to annotate final object (dims=1:20 for multi tissue data and dims=1:10 for peripheral blood data). The resulting merged and normalized matrix was used for the subsequent analysis. To evaluate cluster resolution we utilized clustree before settling on cluster assignment. ADT data was normalized using NormalizeData command with method = ‘‘CLR’’ and a margin of 2.

#### scRNA-seq external data integration

For external sample integration, data was downloaded from the associated repositories for Bugaut et al.^24^ and Wang et al.^25^ For data from Bugaut et al., Thymus NKT, T conventional and MAIT cells were annotated separately by following the publication annotation. SCT Transformation (vars.to.regress=nCount_RNA) was used followed by RunPCA (npcs=20), RunUMAP, FindNeighbors and FindClusters (resolution = 0.6). After annotation, each subset was merged with our human iNKT whole transcriptome dataset. The merged data then followed standard analysis workflow of normalization, variable feature detection, scaled (vars.to.regress = nCount_RNA), followed by PCA, and clustering. IntegrateLayers was then used with CCAIntegration. Finally, RNA assays were split based on their original experiments (orig.ident) and underwent SCTransform, RunPCA, RunUMAP and IntegrateLayers using CCAIntegration (normalization.method=SCT), followed by FindNeighbors and RunUMAP. For data from Wang, all samples underwent standard clustering methods separately as mentioned above. Since some of the data from Wang et al. was derived from mice, we first intersected common features between our human dataset and the Wang et al. dataset and subsetted each matrix on the common features before performing standard processing. Samples again were split by their initial dataset and pre-processed with Normalization, variable gene detection, scaling, PCA, and clustering. IntegrateLayers was then used with CCAIntegration followed by clustering and UMAP generation.

#### Monocle pseudo-time analysis

Cell state decisions and trajectory-based analyses were constructed by Monocle3. All cells from different samples were extracted and imported into Monocle3 for analysis. Parameters for the analysis are consistent with the tutorial by the tool’s developers (http://cole-trapnell-lab.github.io/monocle-release/docs/#constructing-single-cell-trajectories). For the order_cells function, the thymic dense clusters were used as the root nodes. Monocle pseudotime scores were carried over to the original Seurat object for differential gene expression analysis.

#### TEMRA signature

TEMRA related genes were derived from Turk et al.^55^ The signature from the publication includes the following genes *KLRG1*, *KLRF1*, *KLRC1*, *CD244*, *PRF1*, *GZMB*, *GZMH*, *GNLY*.

#### CytoTRACE

CytoTRACE was run using the R implementation (https://cytotrace.stanford.edu/) on the RNA counts matrix (v0.3.3). Resulting values were added to metadata for plotting using Seurat.

#### SCENIC regulon analysis

RNA count matrices were extracted from the subsetted seurat object and run using the parameters of the Docker pySCENIC implementation v0.12.1 (https://pyscenic.readthedocs.io/en/latest/installation.html). Mitochondrial annotated genes and genes with low overall cell counts (<=1% of cells expressing the gene) were filtered prior to running pySCENIC. pySCENIC was run using 50 iterations and AUC scores were recalculated for regulons present in at least 60% of runs and a target frequency >= 15. The final SCENIC activity scores were added to the Seurat object as a new assay and scaled.

#### Non-negative matrix factorization (NMF)

To run NMF we used the R package singlet (v.0.0.99, https://github.com/zdebruine/singlet). NMF gene programs were visualized using the UMAP reduction derived from SCT gene expression data.

#### NMF_17 and NMF_19 gene set score

The NMF_17 gene signature was derived from the following list of genes: *RGS1, CXCR4, CD69, BTG1, TNFAIP3, SARAF, DDX21, KLF6, WHAMM, TSC22D3, JUN, ITM2A, STK17B, PTGER4, IL6ST, FOS, ZBTB10, IRF2BP2, JUNB, FYB1*.

The NMF_19 gene signature was derived from the following list of genes: *CCL5, CD74, GZMK, CXCR4, CST7, NKG7, HLA.B, DUSP2, B2M, RPS26, TXNIP, HLA.A, FYB1, BTG1, TRBC1, SYNE2, SRGN, RUNX3, TMSB4X, GZMA*.

### QUANTIFICATION AND STATISTICAL ANALYSIS

#### Flow cytometry analyses

For comparisons of two groups, P values were determined by paired T tests between groups. For comparisons of more than two groups, P values were determined using paired t tests followed by Holm-Šídák multiple comparisons test. Data are pooled from at least three independent experiments.

#### Differential scRNA expression analyses

For cell-level and cluster-level differential expression, we used the ‘FindMarkers’ or “FindAllMarkers’ Seurat function as appropriate, with min.pct = 0.30 and logfc.threshold=0.3. The resulting differentially expressed genes (DEGs) were then filtered for adjusted p-value < 0.05 and sorted by fold change. All differential expression analyses were carried out using the “SCT” assay after running PrepSCTFindMarkers on object.

## Supplementary Material

1

2

3

4

Supplemental information can be found online at https://doi.org/10.1016/j.celrep.2025.115587.

## Figures and Tables

**Table:** KEY RESOURCES TABLE

REAGENT or RESOURCE	SOURCE	IDENTIFIER
Antibodies		

FC Blocking Reagent, human	Miltenyi	Catalog # 130–059-901; RRID: AB_2892112
BUV395 - Anti-Human CD3 (Clone OKT3 )	Invitrogen	Catalog # 363–0037-42; RRID: AB_2925248
BV421 - Anti-Human CD3 (Clone OKT3 )	Biolegend	Catalog # 300433; RRID: AB_10962690
PerCP-Cy5.5 – Anti-Human CD3 (Clone UCHT1)	Biolegend	Catalog # 300430; RRID: AB_893299
BV421 - Anti-Human CD8β (Clone 2ST8.5H7)	BD Biosciences	Catalog # 742390; RRID: AB_2740746
PE - Anti-Human CD8α (Clone SK1)	eBiosciences	Catalog # 12–0087-42; RRID: AB_1603260
BUV496 - Anti-Human CD8α (Clone RPA-T8)	BD Biosciences	Catalog # 612942; RRID: AB_2870223
BV785 - Anti-Human CD4 (Clone OKT4)	Biolegend	Catalog # 317441; RRID: AB_2561365
FITC - Anti-Human Va24 (Clone 6B11)	Biolegend	Catalog # 342905; RRID: AB_1731930
FITC - Anti-Human Va24 (Clone C15)	Beckman Coulter	Catalog # IM1589; RRID: AB_130878
PE - Anti-Human Vb11 (Clone C21)	Beckman Coulter	Catalog # IM2290; RRID: AB_131325
APC - Anti-Human Vb11 (Clone C21)	Beckman Coulter	Catalog # A66905; RRID: AB_3683578
PE-Cy7 – Anti-Human CD19 (Clone HIB19 )	Biolegend	Catalog # 302215; RRID: AB_314245
AF700 - Anti-Human CD19 (Clone SJ25C1 )	Biolegend	Catalog # 363034; RRID: AB_2616936
APC-Cy7 – Anti-Human CD19 (Clone SJ25C1)	BD Biosciences	Catalog # 557791; RRID: AB_396873
APC-H7 – Anti-Human CD14 (Clone MØP9)	BD Biosciences	Catalog # 560180; RRID: AB_1645464
AF700 - Anti-Human CD14 (Clone M5E2 )	Biolegend	Catalog # 301822: RRID: AB_493747
APC-Cy7 – Anti-Human CD24 (Clone ML5)	Biolegend	Catalog # 311132; RRID: AB_2566347
BV421 - Anti-Human TBET (Clone 4B10 )	Biolegend	Catalog # 644832; RRID: AB_2686976
PE - Anti-Human RORγt (Clone Q21–559 )	BD Biosciences	Catalog # 563081; RRID: AB_2686896
BV421- Anti-Human ISOTYPE (Clone MOPC-21)	Biolegend	Catalog # 400157; RRID: AB_10897939
PE - Anti-Human ISOTYPE (Clone MOPC-21 )	BD Biosciences	Catalog # 557872; RRID: AB_396914
BUV395 - Anti-Human CD45RA (Clone HI100 )	Biolegend	Catalog # 304190; RRID: AB_3097562
PE-Cy7 - Anti-Human CCR7 (Clone 2-L1-A )	BD Biosciences	Catalog # 567314; RRID: AB_2916551
APC - Anti-Human CD45RO (Clone UCHL1 )	Biolegend	Catalog # 304210; RRID: AB_314426
BV421 – Anti-Human CD79a (Clone HM47 )	Biolegend	Catalog # 333519; RRID: AB_3662227
AbSeq Oligo Mouse Anti-Human CD4 (Clone SK3)	BD Biosciences	Catalog # 940001; RRID: AB_2875892
AbSeq Oligo Mouse Anti-Human CD8 (Clone RPA-T8)	BD Biosciences	Catalog # 940003; RRID: AB_2875894
AbSeq Oligo Mouse Anti-Human CD45RA (Clone HI100)	BD Biosciences	Catalog # 940011; RRID: AB_2875902
AbSeq Oligo Mouse Anti-Human CD45RO (Clone UCHL1)	BD Biosciences	Catalog # 940022; RRID: AB_2875913

Biological samples		

Human blood (from platelet pheresis leukoreduction system chambers)	Stanford Blood Center, Our Blood Institute, Oklahoma, ImpactLife	N/A
Human cord blood	Stanford University Binns Program	N/A
Human thymic tissue	Stanford University School of Medicine	N/A
Human bone marrow	Stanford University School of Medicine	N/A

Chemicals, peptides, and recombinant proteins		

Ficoll-paque Plus	Cytiva	Cat# 17144003
Ficoll-paque Premium	Cytiva	Cat# 17544203
Fixable Viability Dye efluor780	ThermoFisher Scientific	Cat# 65–0865-14
Heat Inactivated Fetal Bovine Serum, Qualified (FBS)	Gibco	Catalog # 26–140-079
1x DPBS No Calcium, No Magnesium	Corning	Catalog # MT21031CV
HBSS	Gibco	Catalog # 14025092
DNase I	Sigma Aldrich	Catalog # 10104159001
X-VIVO 10 Serum-free Hematopoietic Cell Medium	Lonza	Catalog # 04–380Q
Human AB Serum	Sigma Aldrich	Catalog # H4522
Bio IVT Human Ab Serum Heat-Inactivated	Fisher Scientific	Catalog # NC9310328
Lonza^™^ BioWhittaker^™^ Cryoprotective Medium	Lonza	Catalog # BW12–132A
4’,6-diamidino-2-phenylindole (DAPI)	Miltenyi	Catalog # 130–111-570

Critical commercial assays		

Anti-iNKT MicroBeads, human	Miltenyi	Catalog # 130–094-842
LS Columns	Miltenyi	Catalog # 130–042-401
CD34 MicroBead Kit UltraPure, human	Miltenyi	Catalog # 130–100-453
EasySep^™^ Release Human PE Positive Selection Kit	StemCell	Catalog # 17654
BD^®^ Hu Single-Cell Multiplexing Kit	BD Biosciences	Catalog # 633781
BD Rhapsody^™^ Targeted mRNA and AbSeq Amplification Kit	BD Biosciences	Catalog # 633774
eBioscience Foxp3 / Transcription Factor Staining Buffer Set	Invitrogen	Catalog # 00–5523-00
Paraformaldehyde, 8% w/v aq. soln., methanol free	Thermo Scientific Chemicals	Catalog # 047347–9M
SepMate^™^−50 (IVD) Tubes	StemCell	Catalog # 85450
Recombinant Human IL-1β (carrier-free)	Biolegend	Catalog #: 579402
Recombinant Human IL-23 (carrier-free)	Biolegend	Catalog #: 574102
Recombinant Human IL-6 (carrier-free)	Biolegend	Catalog #: 570802
Human TGF-β1, premium grade	Miltenyi	Catalog #: 130–095-066
TECINTM (Teceleukin) Recombinant Human Interleukin-2 (rIL-2)	BRB Preclinical Biologics Repository- NCI	N/A
alpha-galactosylceramide [alpha-Gal-Cer; KRN7000]	AdipoGen	Catalog #: AGCN20013M001

Deposited data		

scRNA data	GEO: GSE261557	This Study
Code	https://doi.org/10.5281/zenodo.14984003	This Study
scRNA – External Data Integration	GEO: GSE239558	Bugaut et al. 2023
scRNA – External Data Integration	GEO: GSE130184 and GSE161495	Wang et al. 2022

Software and algorithms		

R (version R4.0.3)	https://www.r-project.org/	RRID:SCR_001905
Seurat	https://satijalab.org/seurat/	RRID:SCR_016341
Matrix (R package)	https://cran.r-project.org/web/packages/Matrix/index.html	
Python	http://www.python.org/	RRID:SCR_008394
pySCENIC	https://pyscenic.readthedocs.io/en/latest/installation.html#command-line-interface	RRID:SCR_025802
Docker	https://www.docker.com/	RRID:SCR_016445
tidyverse (R package)	https://CRAN.R-project.org/package=tidyverse	RRID:SCR_019186
ggplot2 (R package)	https://cran.r-project.org/web/packages/ggplot2/index.html	RRID:SCR_014601
viridis (R package)	https://cran.r-project.org/web/packages/viridis/vignettes/intro-to-viridis.html	RRID:SCR_016696
ggpubr (R package)	https://CRAN.R-project.org/package=ggpubr	RRID:SCR_021139
monocle3 (R package)	https://cole-trapnell-lab.github.io/monocle3/	RRID:SCR_018685
SeuratWrappers (R package)	https://github.com/satijalab/seurat-wrappers	RRID:SCR_022555
AUCell (R package)	https://bioconductor.org/packages/AUCell/	RRID:SCR_021327
SCENIC (R package)	https://github.com/aertslab/SCENIC	RRID:SCR_017247
SCopeLoomR (R package)	https://github.com/aertslab/SCopeLoomR	
utils (R package)	https://cran.r-project.org/web/packages/R.utils/index.html	
pheatmap (R package)	https://www.rdocumentation.org/packages/pheatmap/versions/0.2/topics/pheatmap	RRID:SCR_016418
harmony (R package)	https://github.com/immunogenomics/harmony	RRID:SCR_022206
dplyr (R package)	https://cran.r-project.org/web/packages/dplyr/index.html	RRID:SCR_016708
stringr (R package)	https://stringr.tidyverse.org/	RRID:SCR_022813
clustree (R package)	https://cran.r-project.org/package=clustree	RRID:SCR_016293
gridExtra (R package)	https://cran.r-project.org/package=gridExtra	RRID:SCR_025249
scCustomize (R package)	https://samuel-marsh.github.io/scCustomize/	RRID:SCR_024675
CytoTRACE	https://cytotrace.stanford.edu/	RRID:SCR_022828
singlet (NMF)	https://github.com/zdebruine/singlet	
FlowJo (v.10.10.0)	BD	RRID: SCR_008520
SpectroFlo (v3.0)	Cytek Biosciences	RRID: SCR_019826

## Highlights

- Transcriptome of human iNKT cells from peripheral blood, cord blood, thymus, and bone marrow
- Three distinct transcriptional signatures: naive/precursor, transitional, and Th1/17/NK-like
- Combined Th1/17 pattern similar to human MAIT cells yet distinct from mouse iNKT cells
- Identification of TEMRA-like iNKT cells and two transcriptionally distinct CD8^+^ populations
