# Supplementary material for: Single-cell transcriptomic profiling reveals diversity in human iNKT cells across hematologic tissues
Source: Cell Rep. Author manuscript; Available in PMC 2025 Jul 12. (PMC12255272; doi:10.1016/j.celrep.2025.115587)
Supplement: 1 [file NIHMS2085627-supplement-1.pdf]

Cell Reports, Volume *44*

Supplemental information

Single-cell transcriptomic profiling reveals diversity in human iNKT cells across hematologic tissues

Reyka G. Jayasinghe, Derek Hollingsworth, Nathan C. Schedler, Emily Landy, Chaiyaporn Boonchalermvichian, Biki Gupta, Hao Yan, Jeanette Baker, Beruh Dejene, Kenneth I. Weinberg, Robert S. Negrin, and Melissa Mavers

Figure S1

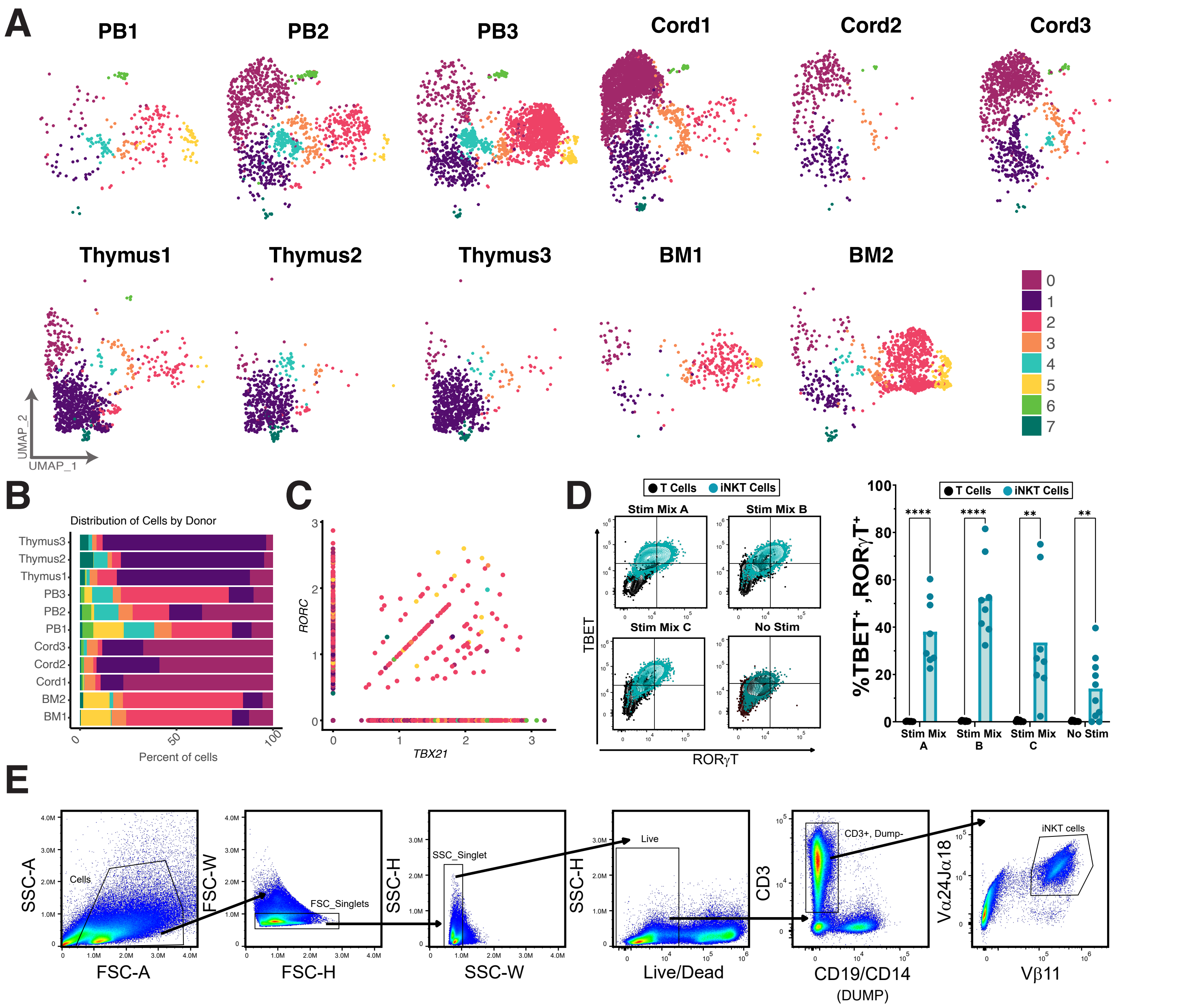

Figure S1. Multi tissue experiment donors and Th1/17 combined signature validation. Related to Figure 1 and Figure 2. A) UMAP representation of iNKT cells derived from multi-tissue samples. Each UMAP is separated by donor and colored by cluster assignment. B) Barplot indicating proportions of iNKT cells restricted to each cluster separated by donor and tissue source. C) Scatter plot showing cells expressing *RORC* and *TBX21*. Each cell is colored by the cluster identity. D) Flow plot and bar graph showing protein validation of TBET and RORγT expression in iNKT cells after stimulation with different cocktails. **$p < 0.001$; ****$p < 0.0001$, determined using paired t tests followed by Holm-Šídák multiple comparisons test. Data are pooled from at least three independent experiments. E) Flow cytometry gating scheme for analysis of transcription factor expression in iNKT cells related to panel D.

Figure S2

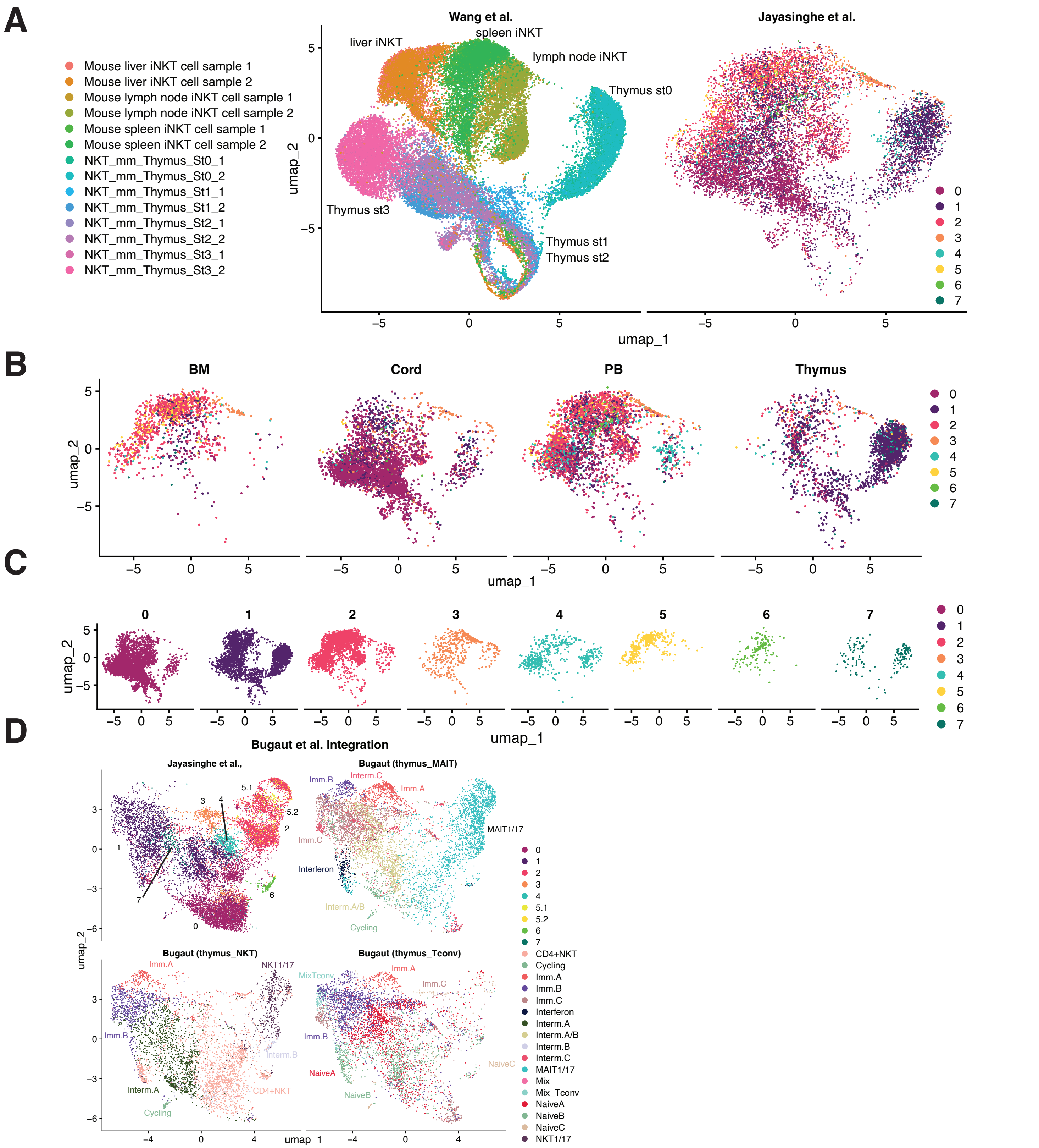

Figure S2. Integration with mouse iNKT cell and human innate lymphocyte datasets. Related to Figure 2 and STAR Methods. A) UMAP representation of cells derived from Wang et al. and the current study. Cells on the left UMAP are colored by sample from Wang et al. Cells on the right are colored by cluster identity. B-C) Integrated UMAP of cells from the current study colored by cluster identity and separated by B) tissue type and C) cluster. D) UMAP representation of integrated object from Bugaut et al. and the current study. UMAP is split by Jayasinghe et al., and each Bugaut donor sample. Cells are colored by the cluster identity for Jayasinghe et al. and for each annotated cell type in Bugaut et al.

Figure S3

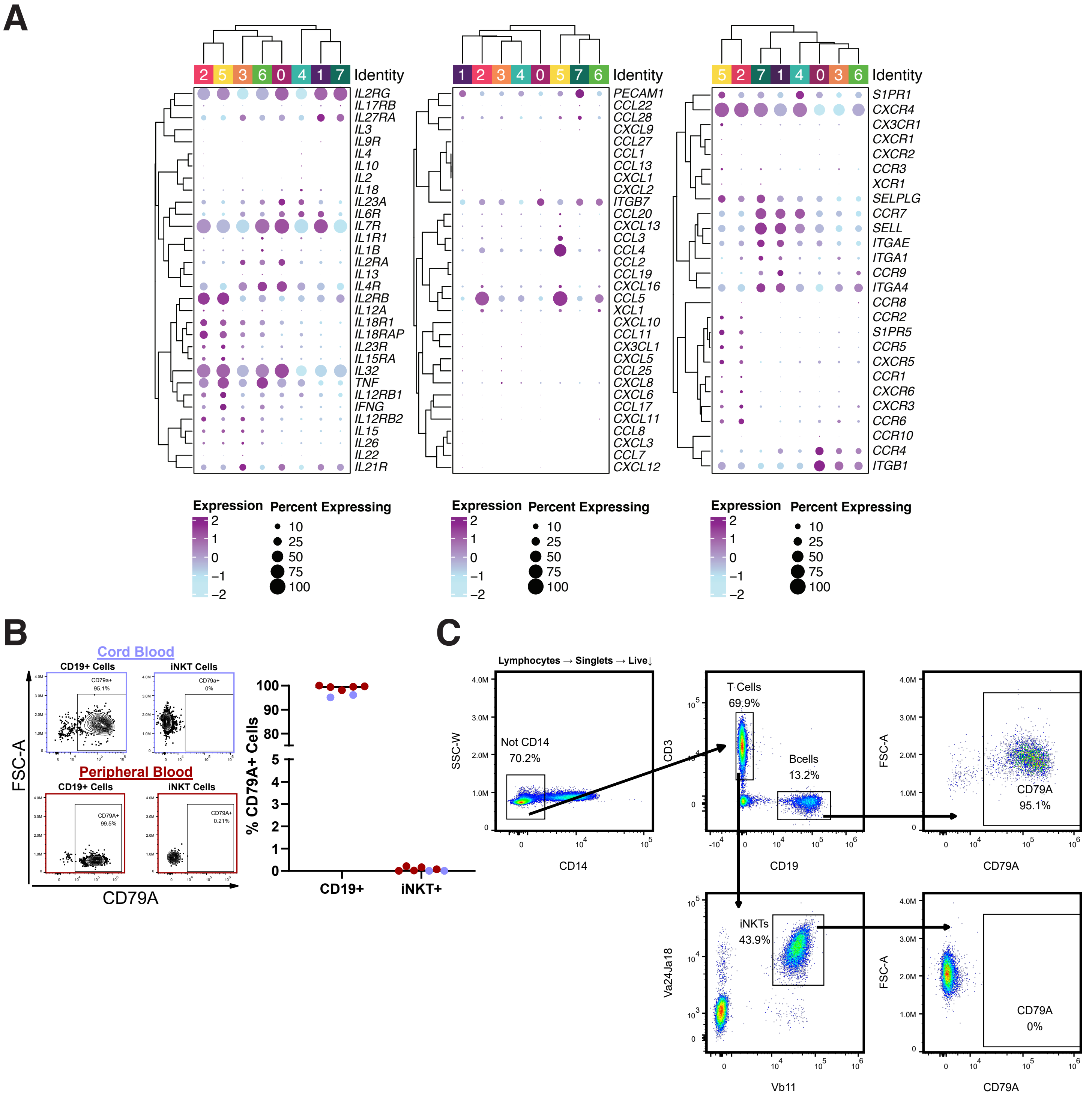

Figure S3. Cytokine, chemokine and chemokine receptor gene and CD79A protein expression patterns. Related to Figures 1 and 2. A) Expression plots of three subsets of genes. Size of dot indicates percent of cells expressing each gene and color indicates the average scaled expression. B) Representative flow cytometry dot plot showing expression of CD79a in CD19+ B cells and iNKT cells (left) and summary flow data of CD79a expression revealing percentages (right) from cord blood (blue) and peripheral blood (red) cells. C) Gating strategy for B cells and iNKT cells.

Figure S4

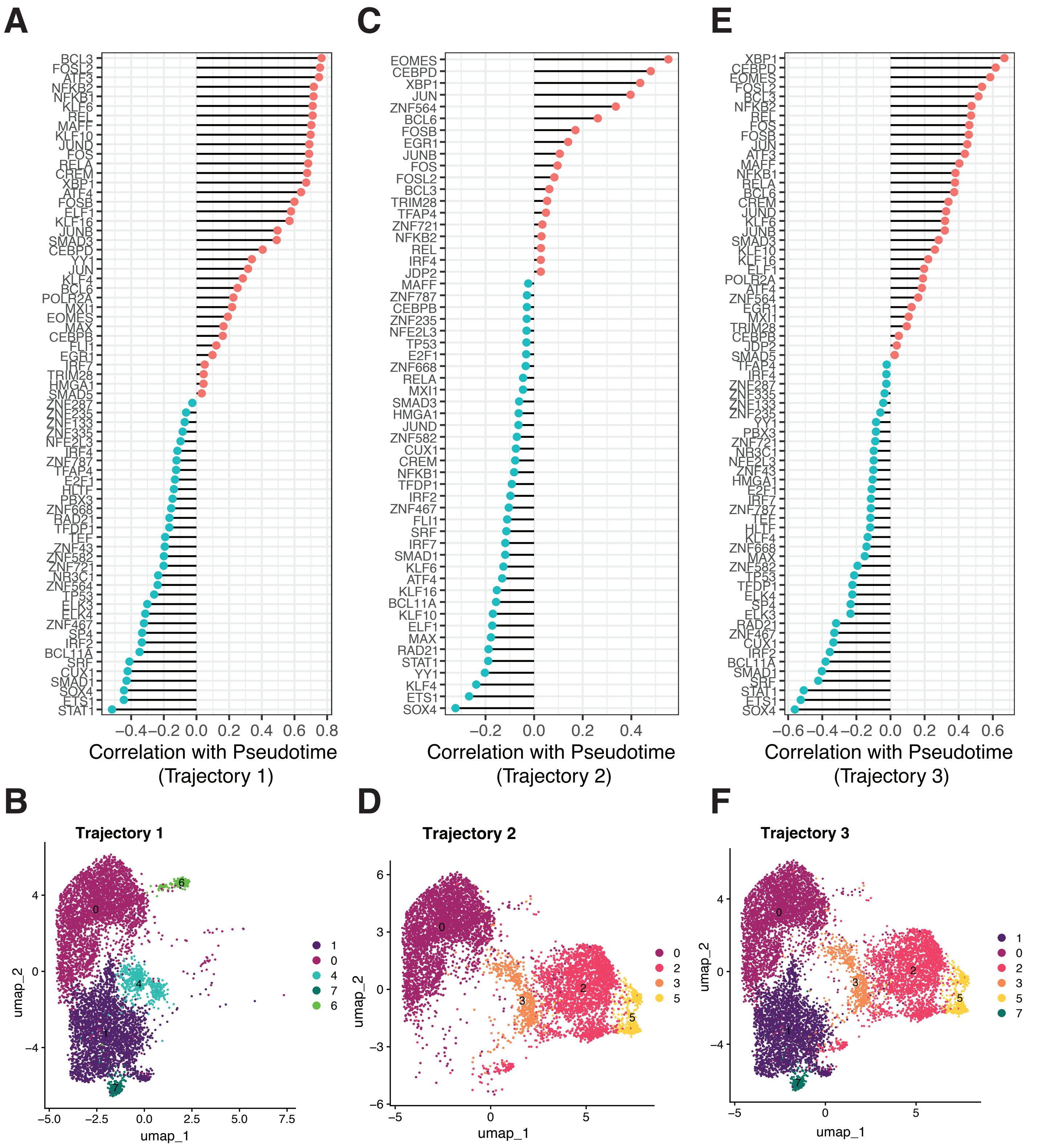

Figure S4. Regulons correlated with pseudotime. Related to Figure 3. A) Regulons correlating with pseudotime across cells in Trajectory 1. B) UMAP of all cells included for the Trajectory 1 regulon and pseudotime correlation analysis. C) Regulons correlating with pseudotime across cells within Trajectory 2. D) UMAP of all cells included for the Trajectory 2 regulon and pseudotime correlation analysis. E) Regulons correlating with pseudotime across all cells. F) UMAP of all cells included for the Trajectory 3 regulon and pseudotime correlation analysis.

Figure S5

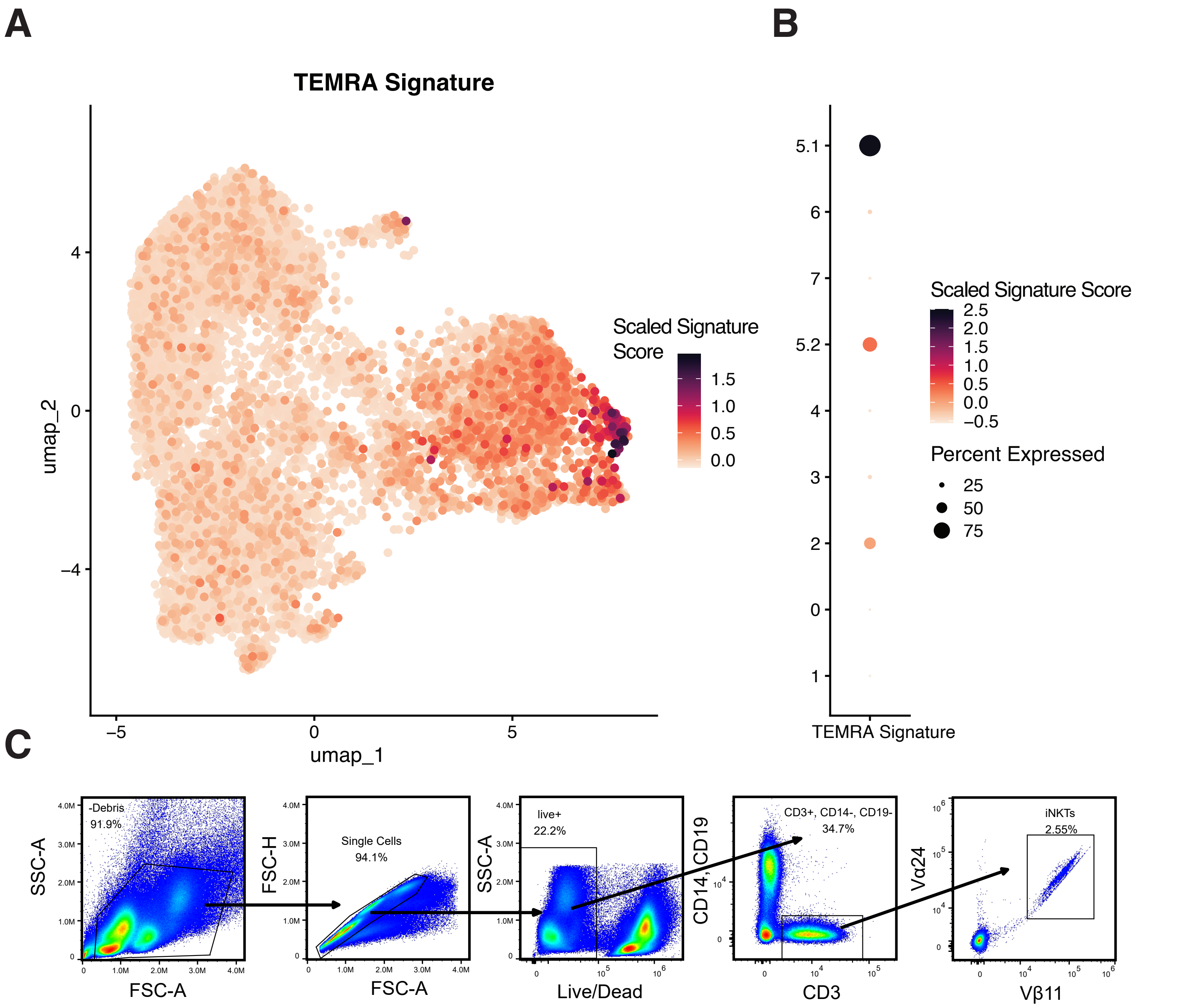

Figure S5. TEMRA gene signature expression and protein validation related data. Related to Figure 4. A) Gene set expression of TEMRA Signature overlaying the iNKT UMAP. Each cell is colored by the scaled signature score of the included gene set. B) DotPlot of the TEMRA Signature separated by cluster identity. Size of dot indicates percent of cells expressing the signature and color indicates scaled signature score. C) Flow cytometry gating scheme for analysis of CD45RA and CCR7 expression in iNKT cells.

Figure S6

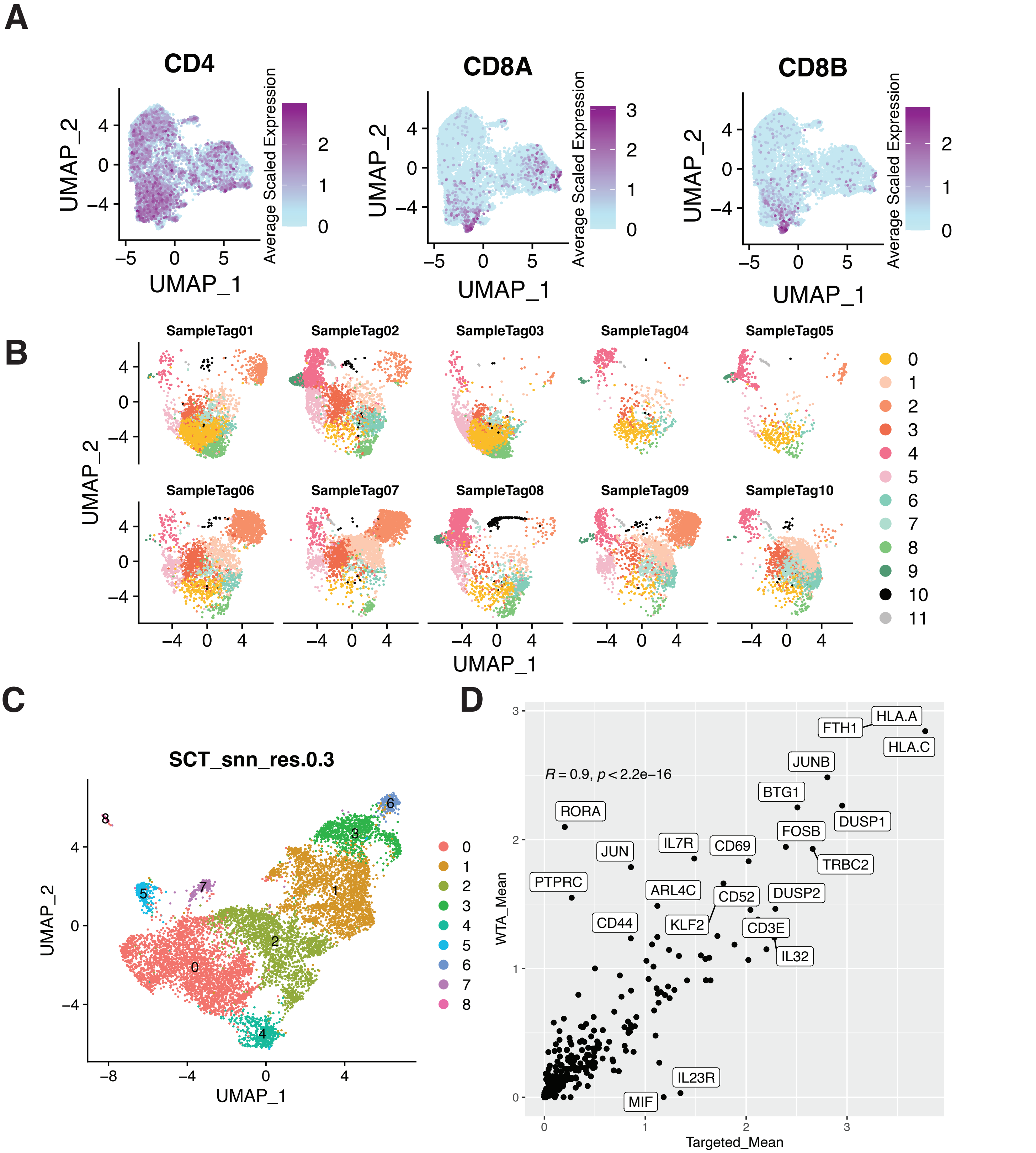

Figure S6. Expression signatures of multi-hematologic tissue and peripheral blood experiments. Related to Figures 4 and 5. A) Feature Plots (multi-tissue experiment) indicating expression of listed genes; colored by average scaled expression. B) UMAP representations of iNKT cells from peripheral blood experiment separated by donor, colored by cluster assignment. C) UMAP of targeted data from the multi-tissue dataset. Each cell is colored by cluster assignment. D) Correlation of the average expression of 382 variable genes overlapping both the whole-transcriptome data and targeted data. Pearson correlation value and p value are indicated on the plot.

Figure S7

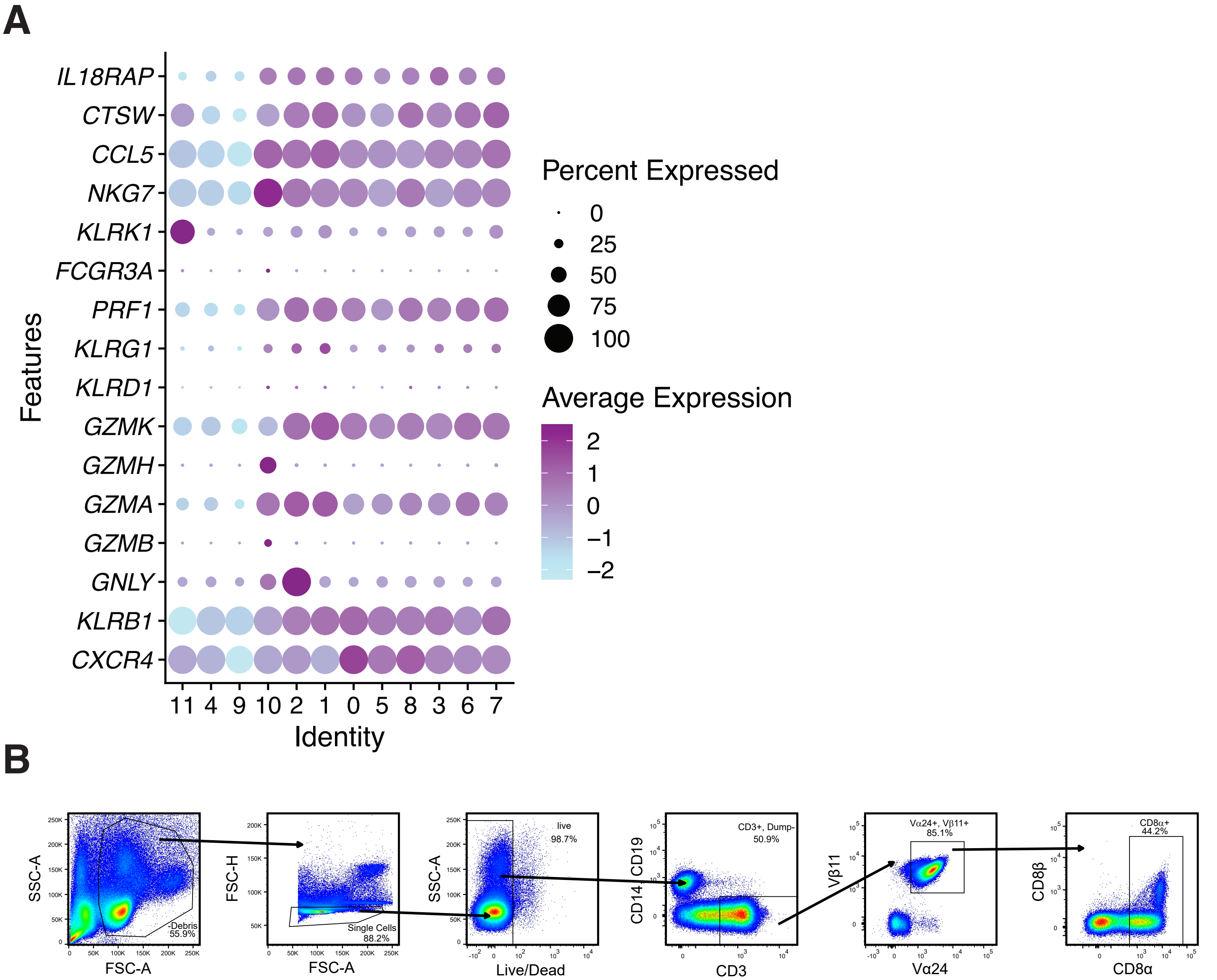

Figure S7. Gene expression in peripheral blood experiment and CD8 protein validation related data. Related to Figures 5 and 6. A) Expression plots of a subset of genes. Size of dot indicates percent of cells expressing each gene and color indicates the average scaled expression. B) Flow cytometry gating scheme for analysis of CD8α and CD8αβ expression in iNKT cells.

Table S1

Supplemental Table 1. Data Quality Metrics.

Tissue_type	Tissue_type_donor	Mean percent.mt	Mean nCount_RNA	Mean nFeature_RNA	Mean nCount_ADT	Mean nFeature_ADT	Number of Cells
BM	BM1	9.78	4857.58	1597.27	688.64	4.59	307.00
BM	BM2	8.49	4942.40	1581.17	1927.65	4.64	1100.00
Cord	Cord1	4.03	5768.12	1715.62	3049.08	4.53	2519.00
Cord	Cord2	4.96	5238.68	1597.62	2551.16	4.42	359.00
Cord	Cord3	4.11	5830.36	1645.37	3173.12	4.52	1019.00
PB	PB1	10.85	5094.41	1492.81	2195.12	4.61	260.00
PB	PB2	6.93	5048.30	1528.92	2701.94	4.56	1133.00
PB	PB3	8.40	4947.22	1507.25	1637.69	4.63	2080.00
Thymus	Thymus1	5.61	6049.92	1932.04	1708.40	4.82	1026.00
Thymus	Thymus2	6.02	5343.13	1767.92	1527.41	4.84	519.00
Thymus	Thymus3	7.13	5620.19	1841.88	1797.75	4.88	717.00

Cluster Label	Number of cells
1	2957
0	3779
2	2562
3	555
4	521
5.2	207
7	156
6	155
5.1	147

Table S1. Statistics for transcriptome datasets. Related to Figure 1 and STAR Methods. Basic statistics for single cell data spanning each donor and tissue type including mean mitochondrial percentage, mean nCount_RNA, mean nCount_ADT, mean nFeature_ADT and number of cells by cluster.
